# The dual role of Actinobacteria in aquaculture: a systematic review of metabolic benefits and detrimental effects

**DOI:** 10.3389/fmicb.2026.1794932

**Published:** 2026-06-10

**Authors:** Claudia Zuluaga-Quintero, Luis Eduardo Diaz Barrera, Luisa Villamil

**Affiliations:** 1 Doctoral Program of Biosciences, School of Engineering, Universidad de la Sabana, Chía, Colombia; 2 Independent Researcher, Bogota, Colombia

**Keywords:** Actinobacteria, aquaculture microbiome, biocontrol agents, off-flavor compounds, quorum sensing inhibition, streptomyces

## Abstract

**Introduction:**

Actinobacteria are ubiquitous and metabolically versatile members of aquatic microbiomes, yet their roles in aquaculture remain under-characterized compared with other microbial groups. This systematic review synthesizes current evidence on their functional duality in farmed fish systems, integrating both metabolic benefits and detrimental effects.

**Methods:**

Studies describing Actinobacterial isolates with at least one laboratory validation were systematically collected and critically evaluated. Methodological approaches were examined to distinguish *in vitro* bioactivity from *in vivo* effects and to assess taxonomic, genomic, and functional characterization.

**Results:**

Only a minority of studies progressed beyond *in vitro* screening, and a critical gap in genomic resolution was identified: almost half lacked molecular identification, and most relied solely on partial 16S rRNA sequencing. *Streptomyces* emerged as the predominant genus, accounting for most reports of both favorable and detrimental effects. Selected *Streptomyces* strains and their postbiotic fractions showed consistent antagonism against major fish pathogens, including *Vibrio harveyi*, *Aeromonas hydrophila*, and *Streptococcus agalactiae*, through quorum-sensing disruption, antibiofilm activity, nutrient competition, and targeted immunometabolic modulation. Conversely, *Streptomyces*, together with *Nocardia* and *Mycobacterium*, have been associated with geosmin and 2-methylisoborneol production, sporadic opportunistic infections, and putative involvement in tetrodotoxin cycling.

**Discussion:**

These outcomes appear highly context-dependent and reflect ecological adaptations rather than intrinsic hazards. By consolidating evidence on both beneficial and detrimental traits, this review highlights the need for genome-resolved taxonomy and ecologically informed screening pipelines to guide the safe and effective integration of Actinobacteria into aquaculture.

## 1 Introduction

In recent decades, aquaculture has become a strategic sector for global food security (Nwuba et al., 2022). However, the industry now faces critical challenges, including the rise of multidrug-resistant pathogens from historical antibiotic use and the need for environmentally sustainable practices that favor higher productivity (Caputo et al., 2023; Thomas et al., 2021). Within this framework, Actinobacteria stand as promising biotechnological agents due to their unique combination of metabolic capacity, ecological adaptability, and functional interactions with aquatic hosts, positioning them as innovative tools to address these challenges (Sunish et al., 2023).

Actinobacteria, currently classified within the phylum *Actinomycetota*, are a bacterial group of significant scientific and biotechnological interest, with applications extending beyond their fundamental ecological role (Ruwandeepika et al., 2022). Indeed, these microorganisms are responsible for producing most of the known natural antimicrobial agents, constituting a critical arsenal in combating bacterial and fungal pathogens. Furthermore, they synthesize a great diversity of other bioactive compounds exhibiting antitumoral, antiviral, and antiparasitic properties, thereby consolidating their central and irreplaceable role in drug discovery and development pipelines (Behera and Das, 2023). Actinobacteria have a broad ecological distribution and are found in a wide range of habitats, including terrestrial soils and aquatic systems (Trenozhnikova et al., 2024). Contemporary metagenomic analyses confirm their persistent integration into the intestinal microbiota of healthy fish, establishing their consistent presence within core piscine microbiomes (Kanika et al., 2025; Vadivel et al., 2021). While strain-specific functional contributions require deeper characterization, their resilience in these commensal communities suggests potential roles in host homeostasis that are poorly understood.

This ecological pattern aligns with core principles of host–microbe symbiosis, in which beneficial bacterial symbionts collectively enhance nutritional metabolism and vitamin biosynthesis, exclude pathogens through ecological niche competition, reinforce intestinal barrier integrity, and modulate immunological responses (Legrand et al., 2020).

Despite all the biotechnological potential, the complete beneficial condition must be weighed against documented adverse effects of some Actinobacteria species that generate secondary metabolites, such as volatile organic compounds, which accumulate in the edible tissues of fish, altering their organoleptic properties and reducing commercial acceptability, particularly in systems with low water exchange rates (Abd El-Hack et al., 2022). Furthermore, under environmental stress, strains considered commensal or beneficial may undergo phenotypic shifts, transforming into opportunistic pathogens capable of inducing chronic infectious processes in cultured organisms (Hashish et al., 2018). This functional ambivalence underscores the necessity for integrated evaluations encompassing microbiological, genomic, functional, ecologic, and productive dimensions (Nam et al., 2023).

This systematic review provides a comprehensive mapping of the research conducted on Actinobacteria within aquaculture systems. This research provides a systematic catalog and critical analysis of the full range of reported effects, encompassing both beneficial applications and detrimental outcomes. In addition, this study examines major methodological trends, such as geographical distribution, origin of isolates, and the formats in which applications have been assessed.

A critical focus is placed on characterizing the distinct research approaches, comparing the objectives and evidence derived from *in vitro* assays versus *in vivo* trials. Furthermore, we assessed the methodological rigor in strain characterization, metabolite reporting, and the availability of genomic sequences in public repositories. By synthesizing the evidence, this work delivers a neutral and integrative perspective on the current state of knowledge regarding Actinobacteria in aquaculture, highlighting critical research gaps requiring future research.

## 2 Materials and methods

### 2.1 Search strategy

This systematic review was conducted following the PRISMA (Preferred Reporting Items for Systematic Reviews and Meta-Analyses) guidelines and the PRISMA checklist is available in Supplementary Table S1 (Moher et al., 2009). A systematic literature search was performed in May 2025 across three electronic databases: PubMed, Scopus, and Web of Science (WOS), without temporal restrictions. The search strategy combined terms related to Actinobacteria and aquaculture using Boolean operators (“AND” and “OR”), as follows:

(“Actinomycetota” OR “Actinobacteria” OR “Actinobacteriota” OR “Actinobacteraeota”) AND (“aquaculture” OR “fish” OR “mariculture” OR “aquatic”)s.

Scopus: TITLE-ABS-KEY(“Actinomycetota” OR “Actinobacteria” OR “Actinobacteriota” OR “Actinobacteraeota”) AND TITLE-ABS-KEY(“aquaculture” OR “fish farming” OR “mariculture” OR “aquatic”).

WOS: TS = (“Actinomycetota” OR “Actinobacteria” OR “Actinobacteriota” OR “Actinobacteraeota”) AND TS = (“aquaculture” OR “fish” OR “mariculture” OR “aquatic”).

PubMed: (“Actinomycetota”[Title/Abstract] OR “Actinobacteria”[Title/Abstract] OR “Actinobacteriota”[Title/Abstract] OR “Actinobacteraeota”[Title/Abstract]) AND (“aquaculture”[Title/Abstract] OR “fish”[Title/Abstract] OR “mariculture”[Title/Abstract] OR “aquatic”[Title/Abstract]).

### 2.2 Eligibility criteria

Articles included in this systematic review were selected based on the following inclusion and exclusion criteria:

*Inclusion criteria:* (a) Original research article, (b) Studies analyzing Actinobacteria within the context of aquaculture, (c) Studies reporting at least one documented beneficial or detrimental effect supported by experimental evidence.

*Exclusion criteria:* (a) Descriptive studies or broad ecological approaches lacking experimental data, (b) Studies lacking sufficient methodological detail (e.g., incomplete datasets, unreported experimental parameters), unpublished technical reports, conference abstracts, graduate theses, non-peer-reviewed manuscripts or grey literature, and secondary literature (e.g., reviews, meta-analyses), (c) Articles published in languages other than English.

**Figure 1:**
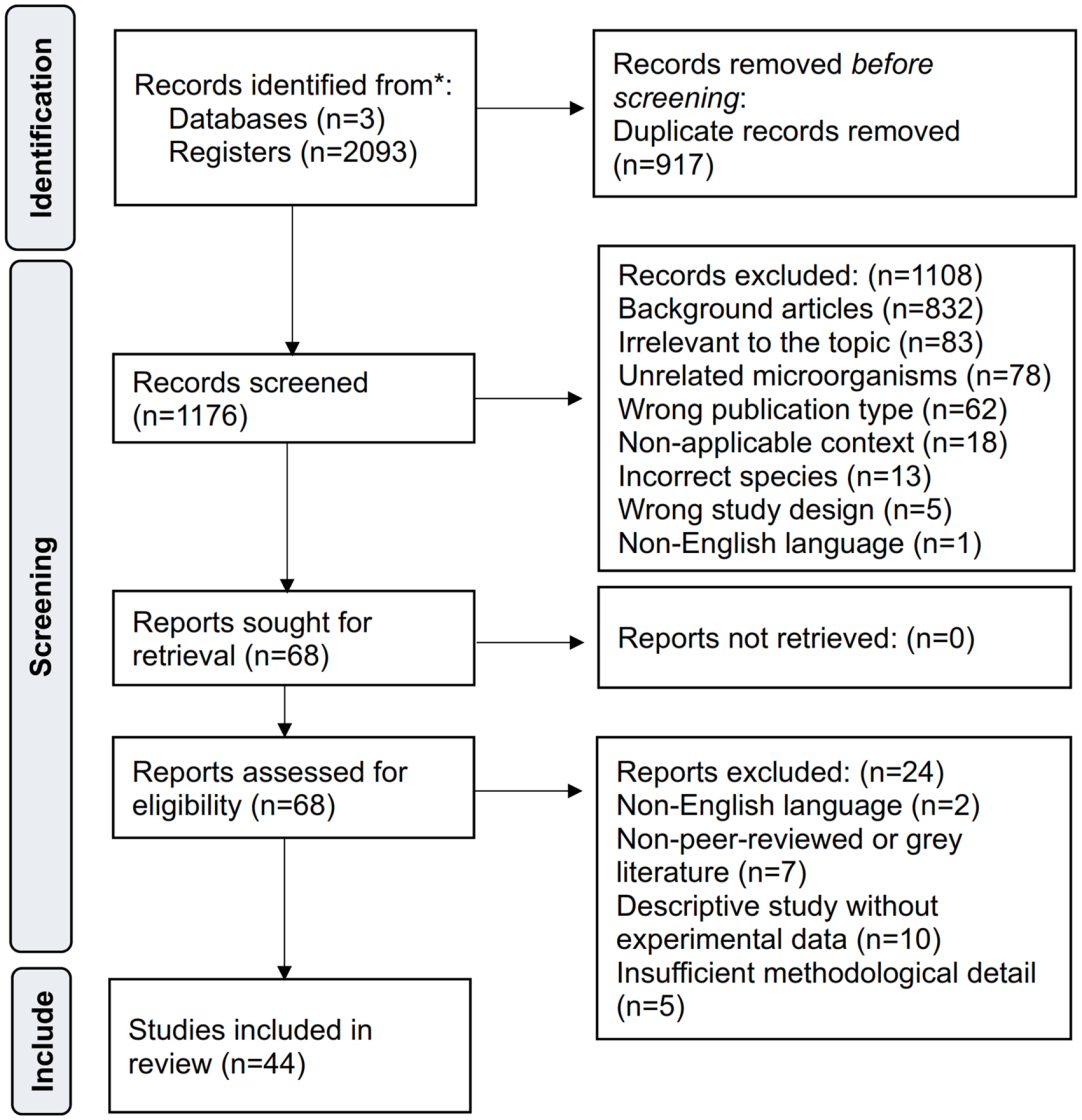
PRISMA flow diagram. Flowchart of systematic literature search according to PRISMA guidelines. Modified from Moher et al. (2009).

Three researchers independently and blindly conducted all stages of the literature selection process. Discrepancies were analyzed and resolved through consensus. A study was considered eligible if at least two reviewers agreed that it met the inclusion criteria. The selection process included identification, duplicate removal, title and abstract screening, full-text eligibility assessment, and final inclusion. During screening, additional exclusion criteria included records that were irrelevant to the research question, focused on unrelated microorganisms or species, or presented inappropriate study designs. The full selection process is summarized in the PRISMA flow diagram (Figure 1).

### 2.3 Data extraction

Studies were initially selected based on evidence regarding the beneficial or detrimental effects of Actinobacteria in aquaculture systems. Additionally, verification was conducted to confirm whether the strains had been sequenced; if so, the corresponding GenBank accession codes were included. Subsequentl, studies were categorized according to the type of experiment performed and divided into two main groups: *in vitro* and *in vivo* assays.

*In vitro* assays: Included studies conducted under controlled laboratory conditions, evaluating the ability of Actinobacteria to inhibit pathogens, produce metabolites with antimicrobial activity, and interact with aquatic environmental components such as nutrients and organic matter. Biochemical and safety assessments were also considered when strains were classified as beneficial.

*In vivo* assays: Analyzed studies assess the direct impact of Actinobacteria on fish. Key parameters included growth rate, survival rate or immune response.

For synthesis purposes, the studies included after the PRISMA selection process were organized into summary tables according to outcome category (beneficial vs. detrimental effects) and type of experimental validation (*in vitro* vs. *in vivo*). Only studies meeting the predefined inclusion criteria and reporting experimentally validated effects were considered for tabulation.

### 2.4 Quality assessment and risk of bias

Given that the evidence base covers both beneficial and detrimental effects, two custom appraisal tools were applied. These tools were adapted from established principles for systematic reviews and risk-of-bias assessment, including Cochrane-style domain-based appraisal approaches and preclinical research guidelines, as well as reliability assessment methods from hazard/risk analysis (Hooijmans et al., 2014; Tran et al., 2021), The tools were applied independently by two reviewers to assess study reliability and bias control.

For *in vitro* studies, a reproducibility checklist (Supplementary Table S2) was used. It evaluated key factors including: strain/compound identification, culture conditions, use of controls, experimental replication, inoculum standardization, quantitative endpoints, dose/exposure characterization, statistical reporting, and the description of key parameters needed for replication. Each item was rated as Yes, No, Unclear, or Not Applicable (NA). A percentage score was calculated as (Yes / evaluable items) × 100, excluding NA items. Studies were then categorized as High, Moderate, or Low quality based on pre-defined thresholds.

For *in vivo* studies, a domain-based risk of bias framework was applied (Supplementary Table S3). This tool assessed specific domains: randomization and allocation concealment, blinding, unit of analysis, handling of incomplete outcome data and exclusions, selective reporting, and other potential biases. Each domain was judged as having Low, Unclear, or High risk of bias. An overall risk-of-bias judgment (Low, Some concerns, or High) was then assigned for each study using pre-specified rules aligned with established guidelines (Hooijmans et al., 2014).

#### 2.4.1 Data synthesis

Studies were grouped first by outcome category (metabolic benefits vs. detrimental effects), and within each category by study type (*in vitro* vs. *in vivo*). Quality/risk-of-bias ratings were used to weight confidence in the findings, prioritizing higher-quality *in vitro* evidence and lower risk-of-bias *in vivo.*

### 2.5 Data analysis and visualization

Phylogenetic analysis was conducted using 16S rRNA gene sequences retrieved from GenBank for selected Actinobacteria strains with reported biological activity. Sequences were aligned using the MUSCLE algorithm implemented in MEGA software. The alignment was manually curated to remove poorly aligned regions and positions with excessive gaps, resulting in a final dataset comprising a shared region of 1,041 bp. The best-fit nucleotide substitution model was determined using the Bayesian Information Criterion (BIC), identifying the Kimura 2-parameter model with a gamma distribution (K2 + G) as optimal. Phylogenetic relationships were inferred using the Maximum Likelihood method with 1,000 bootstrap replicates. *Bacillus subtilis* was used as the outgroup.

Graphical representations were generated using different software tools depending on figure type. The phylogenetic tree was constructed using MEGA12, version 12.0.14, and further edited for visualization purposes. Heatmaps and graphical summaries were generated using RStudio, version 2025.09.0, and Microsoft Excel, version 2,108. Chemical structures were generated using MolView and subsequently edited to improve visual consistency. Final figure assembly, formatting, and labeling were performed using Microsoft PowerPoint.

## 3 Results

### 3.1 Literature review analysis

The literature review identified a total of 2,093 potential studies. Following the removal of 917 duplicate records, 1,176 studies underwent title and abstract screening. During this initial phase, 1,108 records were excluded for not meeting the predefined eligibility criteria (Figure 1). From this screening, 68 articles were selected for a comprehensive full-text assessment. Of these, 24 reports were excluded based on grounds related to methodological rigor and eligibility, as outlined in Figure 1. Finally, 44 studies met all eligibility criteria and were included in the data extraction; the extracted dataset is available as Data Sheet 1.

### 3.2 Quality assessment and risk of bias

Methodological quality of the included *in vitro* studies was evaluated using the prespecified reproducibility checklist (Supplementary Table S2). Overall, 33/42 studies were classified as High quality, 3/42 as Moderate, and 6/42 as Low. Across the lower-rated studies, the most frequent limitations were incomplete reporting of experimental controls, replication, and/or inoculum or exposure standardization, which reduced reproducibility and limited comparability across assays and endpoints.

Risk of bias in the included *in vivo* studies was assessed using the domain-based framework (Supplementary Table S3). Overall, 2/8 studies were judged Low risk of bias, 3/8 presented Some concerns, and 3/8 were rated High risk of bias. The domains most commonly contributing to elevated risk were unclear randomization/allocation procedures, limited (or unclearly reported) blinding, and the experimental unit (fish vs. tank).

These assessments were used to interpret the strength of the evidence across the review. Findings supported primarily by high-quality *in vitro* studies and/or low risk of bias *in vivo* studies were considered more reliable, whereas conclusions based mainly on lower quality or higher risk studies were interpreted with greater caution.

### 3.3 Publication origin and isolation habitats

**Figure 2:**
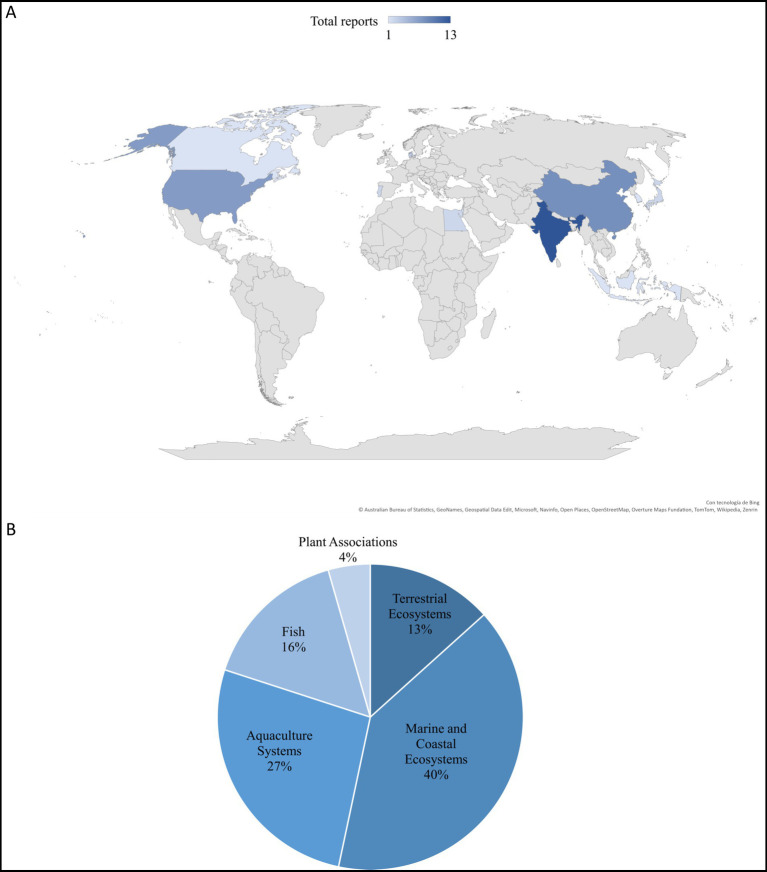
**(A)** World map illustrating countries with reported applications of Actinobacteria. Color intensity corresponds to the number of studies documented per country, with darker shades indicating higher counts. **(B)** Distribution of isolated sources of Actinobacteria with reported effects.

Analysis of the 44 reviewed studies revealed a heterogeneous geographic distribution of Actinobacteria research in aquaculture, with India contributing the highest number of studies with 13, followed by China with 8 and the United States with 7. Denmark contributed 3 studies, while Egypt, Portugal, and Japan each contributed 2 studies each. Canada, South Korea, Indonesia, Israel, Netherlands, France, Switzerland, Iceland, Iran, and the United Kingdom reported 1 study each (Figure 2A).

Actinobacteria were isolated from a wide range of environments relevant to aquaculture systems (Figure 2B). Marine and coastal habitats, including marine sediments, sponges, mangroves, seagrasses, and macroalgae, represented the most frequent isolation sources. Aquaculture sources such as pond water, pond and reservoir sediments, recirculating aquaculture system (RAS) biofilters, and biosolids constituted the second major category, reflecting resident communities directly associated with culture infrastructure. Additional isolates originated from fish-associated niches (skin, gills, gut, and gonads), as well as from terrestrial and plant-associated environments, including soils, river sediments, and rhizospheres of aquatic or coastal plants.

### 3.4 Association patterns between source and application

**Figure 3:**
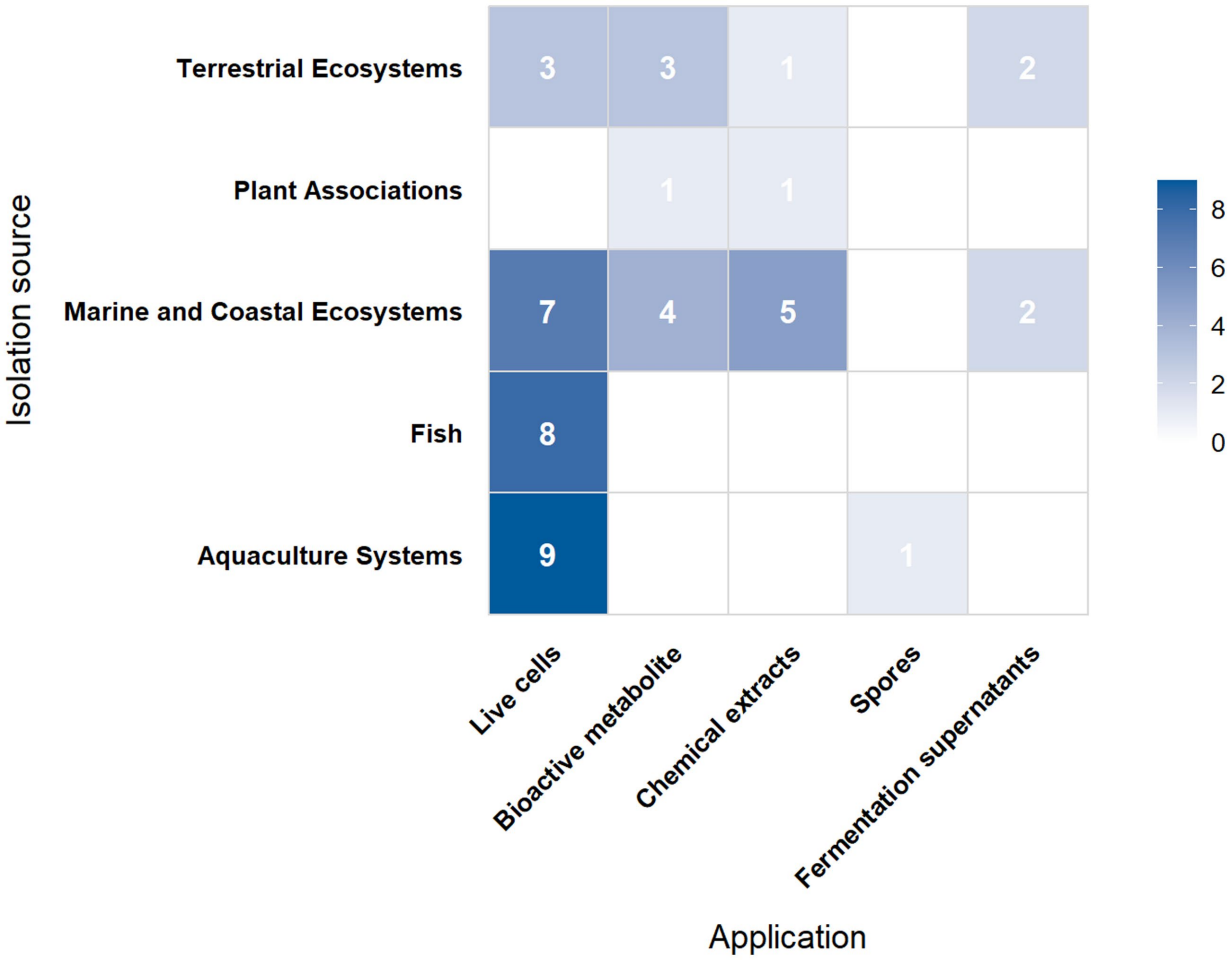
Heatmap showing the frequency of application used to evaluate *Actinobacteria*, stratified by ecological source of isolation. Rows indicate isolated sources (terrestrial ecosystems, marine and coastal ecosystems, plant associations, fish, and aquaculture systems). Columns indicate application (live cells, bioactive metabolites, chemical extracts, fermentation supernatants, gill exposure). Color intensity reflects the relative frequency of each source–application pairing, with darker shades indicating higher usage.

The review revealed that the ecological source of Actinobacteria substantially influences the metabolic and functional characteristics observed. Figure 3 summarizes the relationships between isolation sources and the applications tested, which ranged from live bacterial cells to extracellular derivatives like chemical extracts, fermentation supernatants, and purified metabolites.

Clear patterns emerged linking the environmental source to the type of application. Actinobacteria from aquatic environments, such as aquaculture systems, fish-associated niches, and marine ecosystems, were predominantly tested as live cells. This reflects a research focus on their direct effects within cultural environments. In these live-cell studies, the primary endpoint was often the assessment of detrimental effects, which explains the concentration of research in aquaculture systems was managing risks, such as off-flavor compounds, an operational concern. In contrast, isolates from non-aquatic sources, like terrestrial soils and plant associations, were more frequently evaluated through their extracellular products (e.g., chemical extracts, fermentation supernatants). The goal in these cases is typically the characterization of chemical bioactivity for purposes like drug discovery.

Overall, these patterns delineate an ecological and methodological gradient; briefly, isolates from aquatic environments are typically tested as live cells, whereas those from terrestrial habitats are primarily explored for their metabolic outputs and bioactive potential.

### 3.5 Actinobacteria genus assessed

**Figure 4:**
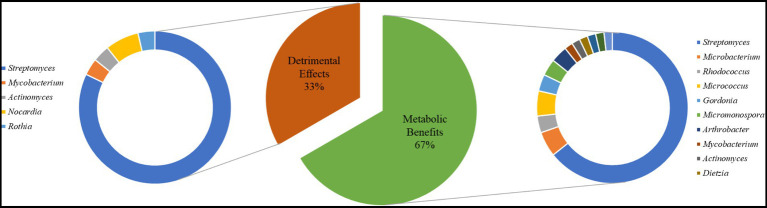
Central pies compare study counts for metabolic benefits vs. detrimental effects; flanking pies show the genus-level composition within each outcome, highlighting the dominance of *Streptomyces* and the long-tail contribution of other genera.

Across the 44 publications studied, taxonomic and functional analyses identified 84 Actinobacterial isolates from 15 genera, enabling their grouping into two major research themes: metabolic benefits and detrimental effects (Figure 4). Research on metabolic benefits (31 studies) was heavily centered on the genus *Streptomyces*, with a primary focus on antibacterial activity. Isolates from genera such as *Microbacterium*, *Dietzia*, and *Rhodococcus*, among others, were reported sporadically, indicating a wide but underutilized phylogenetic reservoir for bioprospecting. Conversely, investigations of detrimental effects (13 studies) were exclusively linked to the synthesis of off-flavor compounds. In this context, *Streptomyces* was the principal agent, with infrequent involvement of *Rothia* and *Nocardia*.

In summary, *Streptomyces* constitute the primary taxonomic and functional node linking beneficial metabolic activities with major detrimental quality problems in aquaculture systems, underscoring the extensive biodiversity and functional versatility of this genus.

### 3.6 Phylogenetic and functional relationships

**Figure 5:**
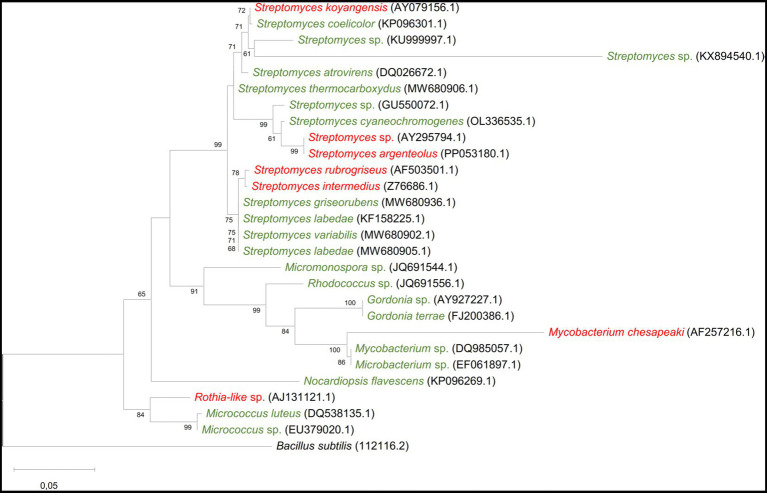
Maximum likelihood phylogenetic tree based on partial 16S rRNA gene sequences of selected *Actinobacteria* strains. Sequences were aligned and curated to a final dataset comprising a shared region of 1,041 bp. The phylogenetic tree was inferred using the Kimura 2-parameter model with a gamma distribution of rate variation among sites (K2 + G). Node support values were calculated using 1,000 bootstrap replicates, and only values ≥50 are shown. *Bacillus subtilis* was used as the outgroup. Strain labels are color-coded according to their reported biological activity: green indicates beneficial effects (e.g., antimicrobial, probiotic, or growth-promoting), while red indicates detrimental effects (e.g., toxin production, off-flavor compounds, or pathogenic potential). Accession numbers are indicated in parentheses.

Across the 44 studies analyzed, approximately 84 Actinobacterial isolates were documented. Of these, 37 have publicly available molecular data, while 47 lack genetic information. Among the sequenced isolates, 35 are represented solely by 16S rRNA gene sequences, and only two isolates, *Streptomyces violaceoruber* CT-F61 and *Streptomyces enissocaesilis* L-82, have complete genome assemblies. Most sequenced isolates belong to the genus *Streptomyces*, whereas non-*Streptomyces* genera such as *Rhodococcus*, *Nocardia*, and *Gordonia* remain largely uncharacterized genetically (Figure 5).

A 16S rRNA phylogenetic reconstruction was performed using 27 isolates with available sequences and experimentally validated effects that met minimum sequence length and alignment overlap criteria. Of these, 21 were classified as beneficial and 6 as detrimental according to their reported biological activity (Figure 5). Beneficial strains clustered predominantly within a core *Streptomyces* lineage and were associated with reported antimicrobial or bioactive activities, including representatives such as *Streptomyces cyaneochromogenes*, *Streptomyces coelicolor*, *Streptomyces griseorubens*, *Streptomyces labedae*, and *Streptomyces* var*iabilis*. In contrast, detrimental strains were mainly associated with aquaculture systems or diseased fish and included geosmin/2-MIB producers such as *Streptomyces rubrogriseus* and *Streptomyces intermedius*, as well as pathogenic species such as *Mycobacterium chesapeaki*. Overall, the isolation environment was associated with the reported functional outcome: strains from marine, coastal, or host-associated sources were more frequently linked to beneficial effects, whereas aquaculture-derived isolates were more often associated with adverse outcomes.

The 16S rRNA-based phylogeny partially reflected this functional dichotomy but lacked the resolution to fully segregate traits. Several *Streptomyces* clades contained both beneficial and detrimental representatives, indicating that functional divergence occurs below the taxonomic resolution of the 16S rRNA gene. Importantly, natural marine sources, host-associated microbiota, and aquaculture systems should be interpreted as distinct ecological contexts. Marine sediments, sponges, and host-associated niches are generally less directly shaped by intensive culture practices, whereas aquaculture systems are managed environments characterized by stocking density, feed input, organic matter accumulation, water exchange, and biofilter or sediment dynamics. These differences may influence the abundance, activity, and functional roles of Actinobacteria.

This pattern suggests that 16S rRNA analysis reflects ecological structuring more reliably than functional specialization. While isolates from marine, coastal, or host-associated sources were often phylogenetically clustered and predominantly associated with beneficial effects, aquaculture-derived isolates, despite sometimes being phylogenetically proximate, showed functional divergence toward off-flavor production or pathogenicity. Resolving this taxonomy–function decoupling will require complete genome data to identify biosynthetic gene clusters and virulence determinants that directly underlie phenotypic outcomes.

### 3.7 Metabolic benefits

The potential of Actinobacteria to confer metabolic benefits in aquaculture was prominent, with 31 out of the 44 reviewed studies (70.5%) reporting beneficial effects. The research approach was predominantly *in vitro*, with all 31 studies including at least one laboratory assay. A critical subset of six studies further validated these findings through *in vivo* experiments, successfully corroborating the beneficial effects.

#### 3.7.1 *In vitro* evidence for metabolic benefits

*In vitro* studies form the foundational evidence for the metabolic benefits of Actinobacteria in aquaculture, with a strong emphasis on their anti-infective potential. The bioactivity of these strains has been systematically evaluated against major aquaculture pathogens. The primary mechanisms identified include direct antibacterial activity, inhibition of biofilm formation, and disruption of quorum-sensing (QS) communication, all of which contribute to disease prevention and reduced reliance on conventional antibiotics (Table 1).

**Table 1:** *In vitro* studies included after the PRISMA selection process reporting anti-infective activity of actinobacteria in aquaculture-related systems.

Actinobacteria strain	Isolation source	Target pathogen(s)	Activity type	Evaluation method	Key results	References
*Streptomyces* sp. (N4/4, N4/10)	Pond sediment	*Pseudomonas* sp., *Aeromonas* sp., *Flavobacterium* sp., *Edwardsiella* sp.	Antibacterial	Agar streak	“Very good” inhibition (*Pseudomonas*); 100% inhibition (*Aeromonas*)	Ali et al. (2013)
*Streptomyces tanashiensis*	Mangrove sediments	*Aeromonas hydrophila*, *Vibrio parahaemolyticus*, *Vibrio alginolyticus*	Antibacterial	Disc diffusion	Zones: 9–32 mm	Chakraborty et al. (2015)
*Streptomyces atrovirens* PK288-21	Rhizosphere of *Undaria pinnatifida*	*Edwardsiella tarda*, *Vibrio anguillarum*, *Vibrio harveyi*, *Lactococcus garvieae*, *Streptococcus iniae*, *Streptococcus parauberis*	Antibacterial	MIC, Disc diffusion	MIC: 15–128 μg/mL (lowest: 15 μg/mL vs. *Streptococcus iniae*)	Cho and Kim (2012)
*Streptomyces aureofasciculus*	Indian mackerel tissues	*Bacillus* sp., *Pseudomonas* sp., *Vibrio* sp., *Aeromonas* sp.	Antibacterial	Cross streaking	Inhibition: *Bacillus* (55%), *Pseudomonas* (45%), *Vibrio* (40%), *Aeromonas* (5%)	Choudhury et al. (2008)
*Streptomyces* sp.	Marine sponge	*Aeromonas hydrophila*, *Serratia* sp., *Vibrio alginolyticus*, *Vibrio harveyi*, *Vibrio parahaemolyticus*	Antibacterial	Disc diffusion	Zones: 10–25 mm	Dharmaraj (2011)
*Streptomyces argenteolus* TMA13	Marine seagrass	*Aeromonas hydrophila*, *Aeromonas caviae*, *Edwardsiella tarda*, *Vibrio harveyi*, *Vibrio anguillarum*	Antibacterial, Anti-biofilm	MIC, MBC, Fluorescence imaging	MIC: 6.25–50 μg/mL; MBC: 6.25–100 μg/mL; Biofilm inhibition: 62–74%	Elumalai et al. (2024)
*Actinomyces* sp. AB5	River nile sediment	*Aeromonas hydrophila*, *Pseudomonas florescens*	Bacteriostatic	Broth microdilution	MIC: 64 μg/mL (both pathogens)	Essawy et al. (2021)
*Streptomyces violaceoruber* CT-F61	Macroalgae (*Codium tomentosum*)	*Tenacibaculum maritimum*, *Aeromonas hydrophila*, *Listonella anguillarum*	Antibacterial	Agar diffusion	Zones: 14–18 mm	Girão et al. (2024)
*Gordonia* spp., *Microbacterium* spp.	Marine sponge (*Erylus discophorus*)	*Aliivibrio fischeri*, *Vibrio harveyi*	Antibacterial	Duetz/Janus systems	Inhibition: 9.1% (*A. fischeri*), 6.1% (*V. harveyi*)	Graça et al. (2013)
*Streptomyces fradiae*	Fish gut	*Aeromonas caviae*, *Aeromonas hydrophila*, *Aeromonas salmonicida*, *Aeromonas sobria*, *Streptococcus agalactiae*	Antibacterial	Agar diffusion	Inhibition: 50–100% (*Aeromonas* spp.); 25–50% (*Streptococcus agalactiae*)	Jami et al. (2015)
*Streptomyces* sp.	Marine sediments	*Vibrio* spp.	Vibriocidal	Agar well diffusion method	Zone of inhibition: 20 mm (VS1); 15–17 mm (VS3)	Kamarudheen et al. (2015)
*Streptomyces enissocaesilis*	Marine sediment	*Pseudomonas* sp., *Vibrio anguillarum*, *Aeromona hydrophila*, *Vibrio parahaemolyticus*, *Vibrio harveyi*	Antibacterial	Agar plug method, MIC/MBC	MIC: 15.6–250 μg/mL; MBC: 62.5–250 μg/mL; Zones: 15–26 mm	Kumaran et al. (2020)
*Arthrobacter bergeri*	Cod rearing wáter	*Vibrio anguillarum*	Antibacterial	Zone of inhibition	Zones: 30–39 mm	Lauzon et al. (2008)
*Streptomyces djakartensis*	Mangrove rhizosphere	*Aeromonas hydrophila*, *Providencia vermicola*, *Vibrio parahaemolyticus*	Antibacterial	Disc diffusion, MIC	MIC: 250 μg/mL (*Aeromonas hydrophila*); Zones: 18–21 mm	Manisha et al. (2019)
*Streptomyces* sp.	Beach sediment	*Vibrio harveyi*, *Aeromonas hydrophila*, *Shewanella putrefaciens*	Anti-biofilm	Biofilm assays on surfaces	Biofilm inhibition: 65.9% (*Vibrio harveyi*); Stability reduction: 71.6% (*Aeromomnas hydrophila*)	Miller et al. (2022)
*Streptomyces* sp. VITNK9	Soil	*Aeromonas caviae*, *Aeromonas hydrophila*, *Edwardsiella tarda*, *Vibrio anguillarum*, *Vibrio harveyi*	Antibacterial	Well diffusion assay	Zones: 14–17 mm (CFS); 18.33 mm (ethyl acetate extract)	Nabila and Kannabiran (2018a,b)
*Streptomyces* sp.	Soil	*Edwardsiella tarda*, *Aeromonas caviae*, *Vibrio harveyi*, *Vibrio anguillarum*	Antibacterial	Well diffusion	Zones: 13–17 mm	Nabila and Kannabiran (2018b)
*Streptomyces* sp. RAUACT-1	Seagrass roots (*Syringodium isoetifolium*)	*Aeromonas hydrophila*, *Bacillus subtilis*, *Serratia* sp., *Vibrio harveyi*, *Vibrio parahaemolyticus*	Antibacterial	MIC/MBC assay	MIC: 128 μg/mL (most pathogens); MBC: 128–512 μg/mL	Ravikumar et al. (2012)
*Micromonospora* spp., *Rhodococcus* spp.	Fish intestine	*Vibrio mimicus*, *Vibrio vulnificus*, *Yersinia ruckeri*	Antibacterial	MIC (serial dilution)	MIC: 110.6 μg/mL (*Vibrio mimicus*)	Sanchez et al. (2012)
*Streptomyces* sp. JF899543, JN049635, JN049634	Marine sponge	*Bacillus* sp., *Bacillus cereus*	Antibacterial	Agar well diffusion method	MIC and MBC: 250 μg/mL	Selvan et al. (2012)
*Micrococcus* sp. C5-9	Coral-associated	*Tenacibaculum maritimum*	Antibacterial	MIC determination	MIC: 12.5 μg/mL	Sharma et al. (2020)
*Streptomyces labedae*	Marine sediments	*Vibrio cholerae*, *Vibrio parahaemolyticus*, *Vibrio alginolyticus*, *Pseudomonas fluorescens*, *Aeromonas hydrophila*	Antibacterial	Agar well diffusion	Zones: 8.66–16.33 mm	Thirumurugan and Vijayakumar (2015)
*Streptomyces albogriseolus*	Marine sediments	*Vibrio cholerae*, *Vibrio alginolyticus*, *Vibrio parahaemolyticus*, *Pseudomonas fluorescens*, *Aeromonas hydrophila*	Antibacterial	Cross-streak plate	Zones: 0.33–17.67 mm	Thirumurugan et al. (2018)
*Streptomyces rutgersensis*	Tilapia gut	*Streptococcus agalactiae*, *Streptococcus iniae*, *Micrococcus luteus*, *Vibrio parahaemolyticus*	Antibacterial	Agar blocks, MIC	MIC: 3.1–25 μg/mL; Zones: 11.4–17.2 mm	Wu et al. (2020)
*Streptomyces albus*	Marine sediments	*Vibrio harveyi*, *Vibrio vulnificus*, *Vibrio anguillarum*	Quorum-sensing inhibition	Crystal violet staining, N-AHL bioassays	Biofilm inhibition: 99.3%; Mature biofilm degradation: 75.6%	You et al. (2007)

Target pathogens, activity type, evaluation method, and key results are summarized for each study.

**Figure 6:**
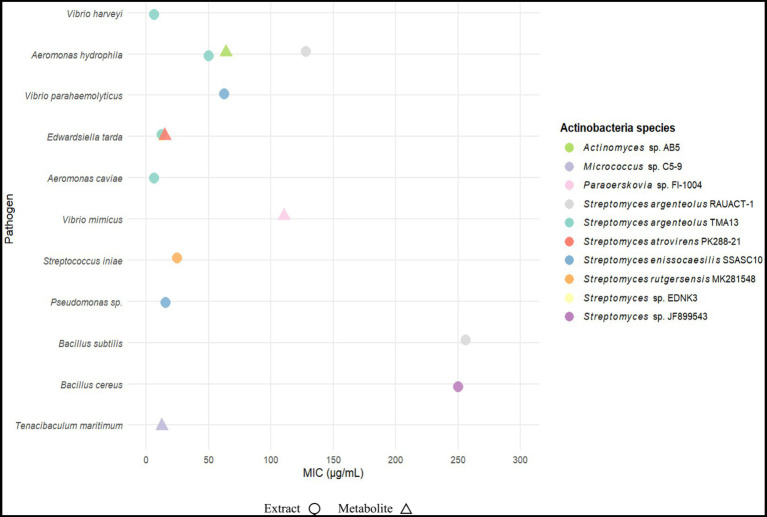
Actinobacterial antibacterial activity (MIC). Scatter of minimum inhibitory concentration (MIC, μg/mL) against each pathogen (*Y*-axis in italics) with MIC on the *X*-axis. Point shape encodes application type (circle = extract; triangle = metabolite), and color encodes the producing actinobacterial species. Each point is a literature-reported measurement; error bars are not shown due to the lack of reported variance.

**Figure 7:**
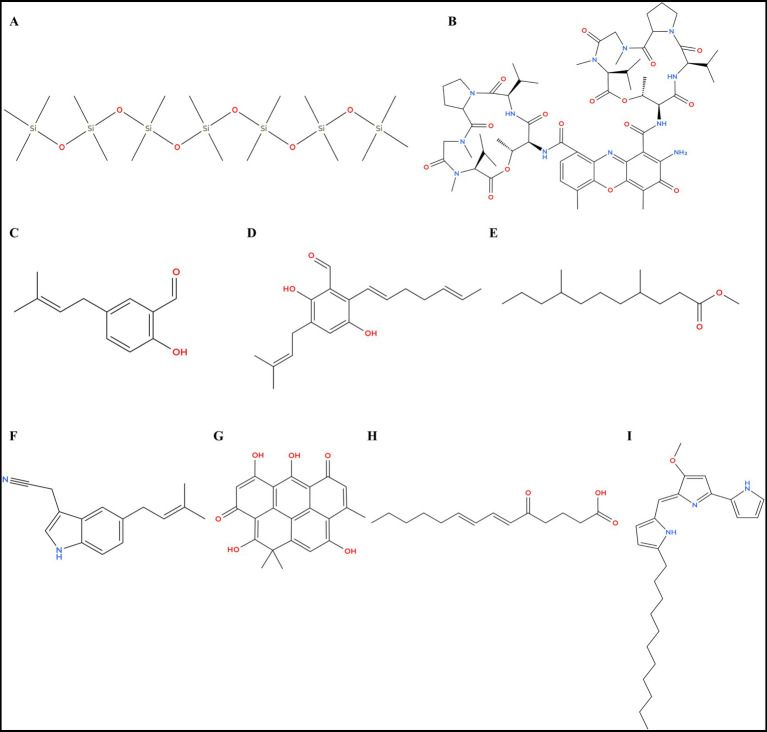
Bioactive compounds isolated from Actinobacteria with beneficial potential: **(A)**, Hexadecamethyl octasiloxane; **(B)**, Actinomycin D; **(C)**, 2-Hydroxy-5-(3-methylbut-2-enyl)benzaldehyde (B1); **(D)**, 2-Hepta-1,5-dienyl-3,6-dihydroxy-5-(3-methylbut-2-enyl)benzaldehyde (B2); **(E)**, Methyl-4,8-dimethylundecanoate; **(F)**, 5-Dimethylallylindole-3-acetonitrile; **(G)**, Heliomycin; **(H)**, (6E,8E)-5-Oxo-6,8-tetradecadienoic acid; **(I)**, Decylprodigiosin.

Antibacterial activity, a consistently reported trait, has been measured using minimum inhibitory concentration (MIC) assays. The compiled data reveal a spectrum of potency, with MIC values ranging from as low as 3.1–512 μg/mL across different Actinobacterial preparations (Figure 6). A clear susceptibility pattern emerges, with prominent activity against Gram-negative pathogens such as *Aeromonas caviae* and *Vibrio harveyi*, often exhibiting high sensitivity (MICs of 6.25 μg/mL) to ethyl acetate extracts of strains like *Streptomyces argenteolus* (Elumalai et al., 2024). In contrast, Gram-positive bacteria such as *Bacillus subtilis* exhibit higher tolerance, with an MIC of 256 μg/mL when exposed to metabolites from *Streptomyces* sp. RAUACT-1. The efficacy is profoundly influenced by the complexity of the preparation; purified secondary metabolites frequently demonstrate superior potency compared to crude extracts, consistent with the structural diversity of bioactive compounds isolated from Actinobacteria (Figure 7). For example, decylprodigiosin from *S. violaceoruber* and benzaldehyde derivatives from *Streptomyces atrovirens* exhibited potent, low MICs of 12.5–15 μg/mL against *Tenacibaculum maritimum* and *Edwardsiella tarda*, respectively (Cho and Kim, 2012; Girão et al., 2024).

However, the observed variation in activity underscores the strain-specific nature of Actinobacterial efficacy. For example, while some strains like *S. argenteolus* and *S. atrovirens* achieved low MICs, others like *S. albogriseolus* showed minimal inhibition against *Aeromonas hydrophila* (Cho and Kim, 2012; Thirumurugan et al., 2018). These disparities highlight the need for systematic screening.

Beyond direct antibacterial activity, several studies documented biofilm disruption and quorum-quenching effects. Specific strains have demonstrated remarkable efficacy in disrupting biofilms, with *Streptomyces albus* reported to inhibit *Vibrio* biofilm formation by over 99% and eradicate pre-formed biofilms by 75.6% through the degradation of N-acyl homoserine lactones (AHLs) (You et al., 2007). Similarly, Actinobacterial extracts have been shown to reduce biofilm biomass of *V. harveyi* and *A. caviae* by 62–74% (Elumalai et al., 2024). This anti-virulence approach, which also includes the inhibition of extracellular enzyme production and bacterial motility, presents a promising strategy for controlling bacterial diseases in aquaculture.

Expanding to compound-level innovation, *Actinomyces* sp. AB5 produced heliomycin, which exhibited synergistic antibacterial activity when combined with biosynthesized silver nanoparticles (AgNPs). The combination yielded FICI (Fractional Inhibitory Concentration Index) values of 0.31 against *Pseudomonas fluorescens* and 0.53 against *A. hydrophila,* values consistent with synergy, resulting in inhibition zones nearly twice those of the controls (Essawy et al., 2021). Such examples illustrate the potential of Actinobacterial metabolites as biotechnological scaffolds for combinatorial or low-dose antimicrobial strategies in aquaculture.

#### 3.7.2 *In vivo* evidence for metabolic benefits

*In vivo* studies confirm the practical potential of Actinobacteria in aquaculture through three main application strategies: live probiotics, purified metabolites or postbiotics, and virulence inhibitors (Table 2).

**Table 2:** Summary of *in vivo* studies included after the PRISMA selection process that met the predefined inclusion criteria and reported experimentally validated effects of actinobacteria on fish or other aquatic organisms.

Species/genus	Strain source	Aplication	Target species	Results	Mechanisms/relevant data	References
*Streptomyces coeruleorubidus*	Soil	Bioactive metabolites	*Oreochromis niloticus*	Immunomodulation: ↑ NO, lysozyme, C3, IgM; ↓ TNF-α, IL-1β, IL-8. - Antimicrobial activity: 86.6% survival inhibition of *Streptococcus agalactiae*. - Hematology: ↑ RBC, Hb, HCT; ↓ leukocytes.	Hexadecamethyl octasiloxane (0.8 mg/L in water). 10 days, 4 replicates.	Abdelaziz et al. (2024)
*Streptomyces cyaneochromogenes*	*Areca catechu* L. (plant-associated)	Bioactive metabolites	*Tenebrio molitor*	At sub-MIC concentrations (12.5–50 μg/mL), actinomycin D acted as a quorum-sensing inhibitor, reducing *Aeromonas hydrophila* virulence traits and improving survival in an infection bioassay	Molecular docking supports interaction with AhyI/AhyR	Zeng et al. (2024)
*Microbacterium oxydans*, *Dietzia maris*, *Rhodococcus qingshengii*	Skin of *Salvelinus fontinalis*	Probiotic	*Salvelinus fontinalis*	- Pathogen elimination: 0 CFU of *Flavobacterium columnare* at 48 h. - Mortality reduction: Efficacy in tanks (2 replicates).	Water administration (10^5^ cells/mL, 21 days).	Boutin et al. (2012)
*Streptomyces* (7 strains)	Marine sponges	Dietary probiotic	*Xiphophorus helleri*	- Growth promotion: +101.53% body weight. - Feed efficiency: FCR = 0.45.	10% biomass in the diet. 50 days, 3 replicates.	Dharmaraj and Dhevendaran (2010)
*Streptomyces enissocaesilis* L-82	Soil	Supernatant/metabolite	*Carassius auratus*	- Antimicrobial activity: 27.5 mm inhibition zone vs. *Aeromonas hydrophila* (MIC: 64-fold dilution). - Innate immunity: ↑ ACP, SOD.	1 × 10^7^ CFU/g in diet. 28 days, 3 replicates.	
*Arthrobacter davidanieli* WX-11	Marine environment	Toxicity assessment	*Caenorhabditis elegans*	- Selective toxicity: 100% mortality in *Caenorhabditis elegans* (72 h). - No effect on *Artemia* sp.	Exposure 1 × 10^7^–1 × 10^9^ cells. 3 replicates.	Neu et al. (2014)

The table includes the bacterial genus or species, isolation source, type of application, target host, main functional outcomes (e.g., antimicrobial activity, immunomodulation, or growth promotion), and relevant experimental details, including administration conditions and, when applicable, identified active compounds.

Live cells or probiotics demonstrated both preventive and therapeutic benefits. In *Carassius auratus* (crucian carp), dietary supplementation with *S. enissocaesilis* L-82 (10^8^ CFU/g feed) for 30 days significantly enhanced growth performance (25.95% weight gain) and immune function, while improving survival rates by 26.67% after *A. hydrophila* challenge (Long et al., 2024). Similarly, a multi-strain *Streptomyces* supplement in swordtail fish feed (10% biomass) remarkably improved body weight by 101.53% with optimal feed conversion (FCR = 0.45) (Dharmaraj and Dhevendaran, 2010). During disease outbreaks, a consortium of seven Actinobacterial strains applied to *Salvelinus fontinalis* (brook charr) rearing water (10^5^ cells/mL) reduced mortality by 54–86% across different genetic families, demonstrating efficacy in the most susceptible lineages (Boutin et al., 2012).

Purified metabolites showed targeted therapeutic effects in *Oreochromis niloticus* (Nile tilapia) challenged with *Streptococcus agalactiae*, waterborne administration of hexadecamethyl octasiloxane (0.8 mg/L) from *Streptomyces coeruleorubidus* increased survival by 33.33% while restoring hematological parameters and modulating immune responses through upregulation of antioxidant genes and downregulation of pro-apoptotic factors (Abdelaziz et al., 2024). The antibacterial compound 5-dimethylallylindole-3-acetonitrile from *S. enissocaesilis* L-82 exhibited direct pathogen inhibition with a 27.5 mm zone against *A. hydrophila* in *C. auratus* for the present case (Long et al., 2024).

Mechanistic and safety studies provided crucial supporting evidence. Fluorescent labeling confirmed the intestinal colonization capability of *S. enissocaesilis* L-82 and its competitive exclusion of pathogens (Long et al., 2024). Actinomycin D from *S. cyaneochromogenes* demonstrated potent quorum-quenching activity, reducing *A. hydrophila* biofilm formation by 73% and increasing *Tenebrio molitor* survival from 23 to 96% through virulence suppression rather than bactericidal action (Zeng et al., 2024). Safety assessments confirmed the absence of adverse effects in key tissues after prolonged administration, supporting the practical application of these strains in aquaculture settings.

### 3.8 Detrimental effects

Among the 44 studies analyzed, 13 (29.5%) reported non-beneficial effects, revealing the dual nature of Actinobacteria as both potential biocontrol agents and sources of detrimental compounds. These adverse effects were categorized into three main types: organoleptic compound production (9 studies), toxic metabolite synthesis (2 studies), and direct pathogenicity (2 studies).

#### 3.8.1 *In vitro* evidence for detrimental effects

*In vitro* studies provided crucial evidence to produce undesirable metabolites through advanced analytical methodologies. Solid-phase microextraction coupled with gas chromatography–mass spectrometry (SPME-GC–MS) was predominantly used to characterize organoleptic compounds, while HPLC analysis detected neurotoxin production (Table 3).

**Table 3:** Summary of *in vitro* studies included after the PRISMA selection process that met the predefined inclusion criteria and reported experimentally validated detrimental effects of actinobacteria relevant to aquaculture.

Species/genus	Strain source	Compound analyzed	Detection method	Key findings	References
*Actinobacteria* sp.	Marine sediment	Tetrodotoxin (TTX)	HPLC	Qualitative TTX production confirmed (0.1 MU/g).	Do et al. (1991)
*Streptomyces roseoflavus*, *Streptomyces thermocarboxydus*	RAS systems	Geosmin, 2-MIB	SPME-GC–MS	MIB > Geosmin in all conditions; anaerobic conditions reduced MIB by 50–70%.	Guttman and van Rijn (2008)
*Streptomyces* spp. 40,006, *Streptomyces intermedius*, *Streptomyces rubrogriseus*	Fishponds	Geosmin, 2-MIB	SPME-GC–MS	Geosmin: 0.12–34.85 ag bacterium^−1^ h^−1^; 2-MIB: ND–15.34 ag bacterium^−1^ h^−1^.	Klausen et al. (2005)
*Actinomycetales* (Group 1), *Myxobacteria* (Group 3)	RAS systems	Geosmin	qPCR (*geoA*) + SPME-GC–MS	Geosmin: 0–21 ng/L; 0.3–0.9% of the microbial population harbored *geoA*.	Lukassen et al. (2019)
*Streptomyces* sp.	Fishponds	2-MIB	SPME-GC–MS	2-MIB: 2.6–5,570 ng/mL.	Pan et al. (2009)
*Streptomyces halstedii*	Pond sediments	Geosmin	GC–MS	0.8–0.9 ug/L after 5 days; no MIB production.	Schrader and Blevins (1993)
*Streptomyces halstedii*	Aquaculture pond sediments	Geosmin	GC–MS	Maximum production with maltose; elevated levels under low phosphorus (<0.2 mM).	Schrader and Blevins (2001)
*Nocardia cummidelens*, *Nocardia fluminea*, *Streptomyces luridiscabiei*	RAS biosolids	Geosmin	SPME-GC–MS	Earthy/musty odors confirmed by olfaction and geosmin quantification.	Schrader and Summerfelt (2010)
*Streptomyces luridiscabiei*	RAS systems	Geosmin, 2-MIB	SPME-GC–MS	Geosmin: 39,765 ng/L (0.16 mg NH₄Cl/L); MIB: 145,042 ng/L (0.16 mg NH₄Cl/L).	Schrader (2020)
*Actinomyces* sp.	*Fugu rubripes* ovaries	Tetrodotoxin (TTX)	HPLC	Low TTX levels detected (0.1 MU/g).	Wu et al. (2005)
*Streptomyces pseudogriseolus* (XHA1), *Streptomyces lanatus* (XHA2), *Streptomyces lavendulae* (XHA3)	Water reservoir	Geosmin, 2-MIB	HSPME-GC–MS	Geosmin: 4.87 ng/mg (XHA1); 2-MIB: 1.27–2.85 ng/mg (XHB3).	Zuo et al. (2010)

Studies include strains isolated from aquatic environments and evaluated using chromatographic, molecular, or toxin-detection approaches. Key findings include geosmin and 2-methylisoborneol (2-MIB) production, tetrodotoxin (TTX) detection, and associations between environmental conditions and metabolite-related responses. SPME-GC–MS, solid-phase microextraction-gas chromatography–mass spectrometry; HSPME-GC–MS, headspace solid-phase microextraction-gas chromatography–mass spectrometry; qPCR, quantitative polymerase chain reaction; MU/g, mouse units per gram; RAS, recirculating aquaculture systems; ND, not detected.

**Figure 8:**
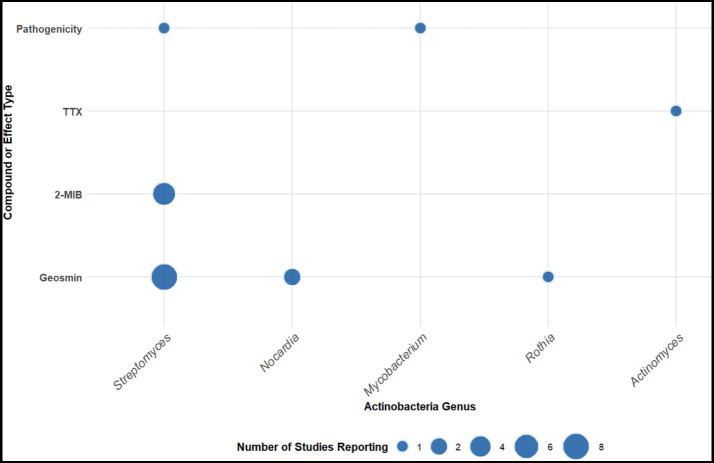
*Actinobacteria* genus-compound/effect associations. Bubble size indicates reporting frequency across studies. 2-MIB, 2-methylisoborneol; TTX, tetrodotoxin.

The production of earthy-musty compounds was extensively documented (Figure 8), with genus-compound association analysis revealing distinct metabolic specialization patterns. *Streptomyces* emerged as the most versatile genus, linked to geosmin (8 studies) and 2-methylisoborneol (2-MIB; 5 studies) production across various aquatic environments. Production levels showed remarkable variation: geosmin concentrations ranged from 47 ng/L in aerobic systems to 39,765 ng/L under nitrogen-limited conditions, while 2-MIB production spanned from 2.6 ng/mL to 145,042 ng/L under similar conditions.

Environmental factors significantly influenced metabolite production, *Streptomyces luridiscabiei* exhibited dramatically increased production of both compounds under low ammonium conditions (0.16 mg/L), while *Streptomyces halstedii* showed enhanced geosmin production under low phosphorus conditions (<0.2 mM) with maltose as carbon source. Nitrogen limitation emerged as a critical regulatory signal, with anaerobic conditions suppressing 2-MIB production in *Streptomyces roseoflavus* by 50–70% compared to aerobic conditions.

Toxic metabolite production in marine-derived Actinobacteria was confirmed by HPLC analysis. Strains isolated from marine sediments and an *Actinomyces* sp. recovered from puffer fish ovaries were found to produce tetrodotoxin (TTX) at levels of 0.1 MU (mouse units, defined as the lethal dose for a standard laboratory mouse) per gram of bacterial biomass (Do et al., 1991; Wu et al., 2005). The consistent detection of TTX in both environmental and host-associated isolates, and the match between their chromatographic profiles and standard TTX, highlights the potential risk of toxin transfer through the marine food chain.

**Figure 9:**
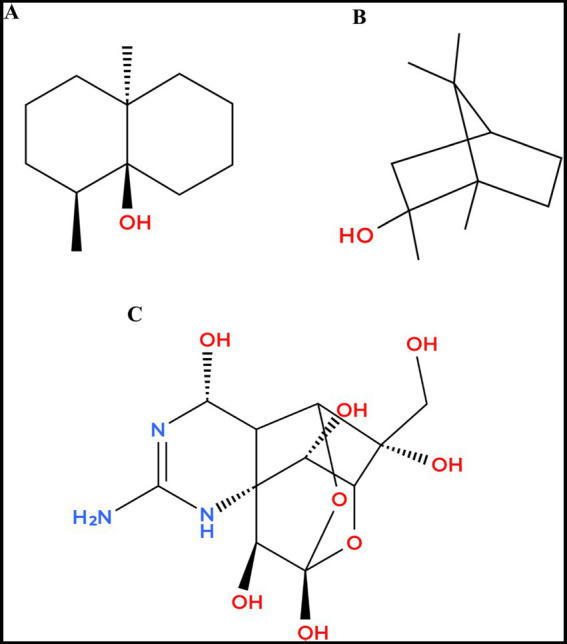
Adverse-effect metabolites and toxins from *Actinobacteria*: **(A)** Geosmin; **(B)** 2-Methylisoborneol (2-MIB); **(C)** Tetrodotoxin (TTX).

Taken together, these studies indicate that *Streptomyces* possess the broadest metabolic capacity associated with geosmin, 2-MIB and TTX (Figure 9). In contrast, other Actinobacterial genera exhibit distinct, specialized ecological niches: *Nocardia* and *Rothia* are linked exclusively to geosmin production, while *Actinomyces* is uniquely associated with TTX synthesis.

#### 3.8.2 *In vivo* evidence for detrimental effects

*In vivo* studies provided direct evidence of the pathogenic potential and toxic effects of certain Actinobacteria in aquatic environments (Table 4). Experimental infections with *M. chesapeaki*, isolated from wild *Morone saxatilis* (striped bass), demonstrated significant pathogenicity through intraperitoneal inoculation. The bacterium induced systemic infections characterized by widespread granulomatous lesions in internal organs and the development of skin ulcers, confirming its role as a primary pathogen in marine fish species (Heckert et al., 2001).

**Table 4:** Summary of *in vivo* studies included after the PRISMA selection process that met the predefined inclusion criteria and reported experimentally validated pathological effects of actinobacteria in aquatic organisms.

Species/genus	Intervention	Target species	Dose/concentration	Observed effects	References
*Mycobacterium chesapeaki*	Intraperitoneal inoculation	*Morone saxatilis* (striped bass)	–	Internal organ granulomas and skin ulcers.	Heckert et al. (2001)
*Streptomyces griseus*	Spore/exudate exposure	*Oncorhynchus mykiss*, *Cyprinus carpio*, *Abramis brama*	1 × 10^5^–1 × 10^7^ spores/Ml	*Oncorhynchus mykiss*: Gill lamellae fusion, hyperplasia. *Cyprinus carpio*: Loss of gill microridges. *Abramis brama*: High mortality.	Lewis et al. (2009)

The table summarizes host species, pathological manifestations, and relevant experimental context, including species-specific lesions such as multiorgan granulomas and gill alterations.

Complementary studies with *Streptomyces griseus* revealed substantial gill pathology in multiple freshwater fish species. Controlled exposure experiments using spore suspensions and bacterial supernatants (1 × 10^5^–1 × 10^7^ spores/mL) over 4 days induced significant histological damage across all tested species: *Oncorhynchus mykiss*, *Cyprinus carpio*, *Abramis brama*, and *Rutilus rutilus*. The observed pathology included lamellar fusion, epithelial hyperplasia, and loss of micro ridging, consistent with an acute gill stress response. The severity of lesions and mortality rates exhibited marked species-specific variation, with *O. mykiss* demonstrating the highest susceptibility, followed by European bream (*A. brama*), which also showed significant mortality (Lewis et al., 2009).

These findings highlight the critical importance of comprehensive strain-specific evaluation before considering Actinobacterial applications in aquaculture systems. The significant influence of environmental conditions on metabolite production, combined with the demonstrated potential for direct pathogenic effects in specific Actinobacterial species, underscores the necessity for rigorous biosafety assessments.

## 4 Discussion

Actinobacteria, renowned for their metabolic versatility and capacity to synthesize bioactive compounds, exhibit dual roles in aquatic ecosystems: while certain strains act as opportunistic pathogens or producers of undesirable odors, others serve as essential symbionts or sources of antimicrobial molecules. This duality is closely linked to their ecological and geographic origins, factors that shape their interactions with hosts (Barka et al., 2015; Zhang et al., 2022). Heterogeneity in their distribution and colonized habitats provides critical insights into their impact on aquaculture, a rapidly expanding sector where balancing health and disease is paramount.

The geographic distribution of research on Actinobacteria in aquaculture reveals a striking asymmetry, with a pronounced concentration in Asian nations, particularly China and India. This regional focus aligns directly with their status as global aquaculture leaders, where the sector represents a fundamental socioeconomic pillar (FAO Fisheries & Aquaculture, 2022). The high number of studies from these regions likely reflects a targeted research response to the pressing challenges of disease management and production optimization in intensive, large-scale farming systems (Stentiford et al., 2017). However, this distribution map exposes critical knowledge gaps that mirror significant global production centers. Major aquaculture producers in Europe, such as Norway, a world leader in salmonid production, and in Latin America, notably Chile, a dominant force in salmon and trout farming, are conspicuously underrepresented in the literature about applied Actinobacterial research (Ceballos-Concha et al., 2025; Misund et al., 2023). Similarly, Ecuador, a top global exporter of shrimp, and other Central American nations with extensive aquaculture operations, show a near-total absence of studies. This geographic disparity underscores substantial untapped potential. The investigation of native Actinobacterial strains within the unique microbiomes, environmental conditions, and predominant pathogens of these understudied yet high-output regions represents a crucial and largely unexplored frontier for bioprospecting and sustainable innovation.

The diversity of habitats from which beneficial Actinobacteria have been isolated, ranging from marine sediments and mangrove rhizospheres to the fish gut microbiota itself, highlights their wide ecological adaptability and underscores their significant promise for aquaculture applications. Previous studies demonstrate that marine-derived strains from sponges and corals produce secondary metabolites with potent antifungal and antibacterial activities, traits often associated with coevolution with sessile hosts that modulate their bioactive profiles (Jackson et al., 2018; Siro et al., 2022). Critically, the consistent isolation of indigenous Actinobacteria from the microbiome of healthy fish (Boutin et al., 2012; Jami et al., 2015) suggests a natural, pre-adapted role in host-associated microbial communities. This intrinsic compatibility positions them as candidates for probiotic applications. Given their proven efficacy in complex ecological contexts, exploring the strategic transfer of these strains, particularly those already native to fish microbiomes, into aquaculture systems represents a logical and promising next step. Such an approach could harness their antagonistic potential to suppress ubiquitous pathogens like *V. harveyi* and *A. hydrophila*, thereby enhancing disease management strategies (James et al., 2023; Mondal and Thomas, 2022).

Beyond geography, the literature shows a wide range of application modalities for Actinobacteria, each with distinct mechanistic advantages. The use of live cells as probiotics relies on their ability to establish and maintain populations within the host microbiome or persist in the rearing environment, where they exert sustained effects through the continuous production of antimicrobial metabolites, competition for nutrients and space, and the exclusion of pathogenic microorganisms (Calcagnile et al., 2024). In parallel, evidence is mounting for postbiotic derivatives or cell-free supernatants and purified bioactive metabolites that deploy the molecular effectors responsible for antibacterial, antibiofilm, and quorum-quenching activities, without introducing viable cells (Sudhakaran et al., 2022). Postbiotics offer practical benefits, including simpler storage, standardization, dosing, and quality control, while reducing ecological and biosafety concerns associated with live microbial release. In practice, the choice between probiotic and postbiotic approaches is context-dependent, shaped by the target species, production system, and the physiological traits of the selected Actinobacterial strain.

Nevertheless, isolating Actinobacteria in controlled aquaculture environments (e.g., ponds, recirculating aquaculture systems, RAS) presents challenges. In controlled environments, certain strains may acquire antimicrobial resistance determinants through horizontal gene transfer, such as class 1 integrins, underscoring the need for strict biosafety screening during strain selection (Dias et al., 2021). Nevertheless, successful colonization of fish microbiota, documented in the gills of *Sparus aurata* and the intestinal tract of *O. mykiss,* demonstrates that some Actinobacteria possess specific adaptive traits. These include FimA-like surface proteins that mediate adhesion to epithelial tissues and a pronounced tolerance to physicochemical fluctuations in pH and salinity. Such attributes can be exploited through the deliberate selection of marine or terrestrial strains already validated in other biological systems, followed by metabolic preconditioning in aquaculture matrices to enhance compatibility and performance (Abdel-Aziz et al., 2021; Butt et al., 2024; Chang et al., 2024; Huang et al., 2024).

The ecological origin of each isolate plays a decisive role in shaping its biosynthetic repertoire. Actinobacteria from environments characterized by close host–microbe associations, pronounced abiotic stress, or high chemical complexity, such as marine sponges, mangroves, and deep-sea sediments, exhibit broader metabolic diversity and greater secondary-metabolite potential than those from more benign habitats (Gozari et al., 2021; Rajivgandhi et al., 2024). Consequently, prioritizing ecologically specialized niches for strain prospecting, coupled with rigorous adaptation assessments, offers a rational pathway for identifying metabolically rich and environmentally resilient Actinobacteria suitable for aquaculture applications while mitigating the risk of resistance dissemination.

On the other hand, regarding taxonomic and functional analysis of Actinobacteria, it was found a scarce information on whole genome sequences, with 56% of studies reporting no basic genomic data, and none incorporated multi-omics (transcriptomics, metabolomics, or proteomics). Among those that did provide DNA sequences, 41% relied only on partial 16S rRNA. While 16S has underpinned microbial taxonomy for decades, it provides poor resolution for Actinobacteria with high genomic plasticity, notably *Streptomyces* and *Mycobacterium*. Near-identical 16S sequences (>97–99% identity) can correspond to organisms with markedly different functional and biosynthetic capacities, a mismatch driven by evolutionary conservation, homoplasy, and the prevalence of horizontal gene transfer in this phylum (Antony-Babu et al., 2017; Gupta et al., 2018). Practical consequences include misidentification and mismanagement: *Mycobacterium marinum* shares 99.6% 16S identity with the human pathogen *Micrococcus ulcerans*, yet they differ in plasmids encoding mycolactones that are central to virulence and diagnostics (Combe et al., 2023). Similarly, in *Micrococcus*, 16S fails to separate fish-pathogenic *Micrococcus luteus* from beneficial *Micrococcus yunnanensis* despite relevant genetic distinctions (Huang et al., 2019; Majhi et al., 2024; Zhu et al., 2021).

Our phylogenetic reconstruction reflects these limits. Actinobacterial isolates tended to cluster by ecological niche (for example, sponge-associated, mangrove-derived, or sediment-derived lineages), indicating environmental filtering and shared selective pressures. However, metabolic benefits versus the detrimental effects of phenotypes did not form discrete clades. In other words, ecological origin showed a signal, but functional outcome did not map cleanly onto 16S-level phylogeny. This pattern is consistent with the idea that medically and aquaculture-relevant traits, such as adhesion, stress tolerance, antagonism, quorum-quenching, or toxin production, are often encoded on mobile or rapidly evolving genomic regions that 16S cannot resolve.

To move beyond these constraints, whole-genome approaches are increasingly used to define species and to connect genotypes to phenotypes. Metrics such as average nucleotide identity (ANI), digital DNA–DNA hybridization (dDDH), and comparative profiling of biosynthetic gene clusters (BGCs) provide the necessary taxonomic and functional resolution (Hu et al., 2022). In Actinobacteria, this has already prompted reclassifications; *Streptomyces costaricanus*, *Streptomyces murinus*, and *Streptomyces phaeogriseichromatogenes* collapsed into a single species, whereas *S. graminearus* remained distinct based on PKS and NRPS architectures (Saygin, 2021); *Nocardia* taxa were consolidated into four groups; *Nocardia soli* and *Nocardia cummidelens* merged into *Nocardia salmonicida*, and using whole-genome data and dDDH thresholds alongside phenotype, including resistance traits (Tamura et al., 2018). Within our dataset, whole genomes were reported only in a subset of studies, but where available, they allowed direct linkage of genes of interest to observed activities. For example, *Streptomyces lateritius* Z1-26 carried 28 BGCs (including six PKS, four NRPS, and bacteriocins) consistent with broad-spectrum antibacterial activity against fish pathogens (Yang et al., 2023). Likewise, genome-guided inference helps to explain anti-virulence phenotypes, such as quorum-sensing interference and antibiofilm effects attributed to actinomycins and related metabolites (Bai and Rai, 2022; Zeng et al., 2024). The priority is therefore clear: expand genome-resolved sampling and integrate even minimal multi-omics to connect ecological niche, taxonomic identity, and actionable traits in aquaculture.

Actinobacteria, particularly *Streptomyces*, exert direct antimicrobial effects and community-level modulation that is evident *in vitro*. Multiple strains suppress canonical virulence behaviors in fish pathogens by inhibiting biofilm formation and quorum sensing, thereby weakening colonization, toxin production, and coordination of resistance. For example, *S. cyaneochromogenes* RC1 reduced *A. hydrophila* biofilm by 73% and down-regulated the quorum-sensing regulator ahyI by 97%, consistent with an anti-virulence mode of action (Zeng et al., 2024). Similar effects have been attributed to actinomycins and related metabolites that interfere with transcriptional activation of pathogenicity genes without imposing strong bactericidal selection (Bai and Rai, 2022).

When translated to animals, these signals align with measurable protection; prophylactic exposure of *O. niloticus* to metabolites from *S. coeruleorubidus* (e.g., 0.8 mg L^−1^ hexadecamethyl octasiloxane) before *S. agalactiae* challenge increased survival by 86.6% and counteracted infection-associated immunometabolic disruption by restoring antioxidant defenses (SOD, CAT), reducing pro-inflammatory mediators (IL-1β, TNF-*α*), limiting NF-κB activation, and modulating apoptosis via the Bcl-2/Bax axis (Abdelaziz et al., 2024). Likewise, dietary inclusion of *S. enissocaesilis* L-82 at 1 × 10^7^ CFU g^−1^ enhanced growth and innate immunity in *C. auratus*; indole derivatives such as 5-dimethylallylindole-3-acetonitrile likely engage nuclear receptors (PPAR-*γ*), providing a plausible route from anti-virulence or antimicrobial chemistry to host immunometabolic stabilization (Gim et al., 2018; Yang et al., 2017).

Only seven studies (16%) progressed from *in vitro* screening to *in vivo* testing, and most trials were conducted under conditions that do not reflect the multi-stressor reality of commercial farms. To address this gap, future research should evaluate probiotic and postbiotic formulations under production-relevant settings, incorporate robust dose response and timing designs that distinguish prophylactic from therapeutic use, and include integrated endpoints such as growth, survival, microbiome dynamics, and water quality (Bai and Rai, 2022; Zeng et al., 2024). In parallel, the marked metabolic diversity among *Streptomyces* and related genera supports a tailored approach: some strains provide broad-spectrum pathogen inhibition, whereas others show more specific anti-virulence or growth-promoting effects (Boutin et al., 2012; Chakraborty et al., 2015; Miller et al., 2022). Selecting strains and delivery formats that fit the target pathogens, farming system, and host species is essential. Live cells are preferable when colonization is beneficial, whereas cell-free extracts are better when precise dosing and quality control are required, providing a practical basis for reliable Actinobacteria-based effects in aquaculture.

The role of Actinobacteria in aquaculture may be associated with the species-specific metabolism. It is well known that these microorganisms are considered biofactories of metabolites with variable biological activities. In this context, the secondary metabolites identified to date, including actinomycin D, hexadecamethyl octasiloxane, and decylprodigiosin, illustrate how Actinobacteria translate ecological competition into chemically diverse strategies relevant for aquaculture applications (Caicedo-Montoya et al., 2021). Despite their distinct structures, these molecules converge functionally by targeting key determinants of pathogen: actinomycin D interferes with DNA-dependent transcription and quorum-sensing regulation, thereby reducing virulence factor expression and biofilm formation (Bai and Rai, 2022; Zeng et al., 2024); hexadecamethyl octasiloxane disrupts membrane integrity and inhibits bacterial growth, although its classification as a bioactive metabolite requires careful consideration given the persistence and bioaccumulation concerns associated with siloxanes (Abdelaziz et al., 2024; Cantu and Gobas, 2024); and decylprodigiosin combines iron chelation with membrane and enzymatic disruption, limiting access to an essential micronutrient while impairing cellular processes (Girão et al., 2024; Ma et al., 2025). Taken together, these findings suggest a coherent functional pattern in which Actinobacterial metabolites act through complementary bactericidal and anti-virulence mechanisms, supporting their use as postbiotic candidates for controlling aquatic pathogens, if potency, stability, and environmental safety are rigorously validated.

**Figure 10:**
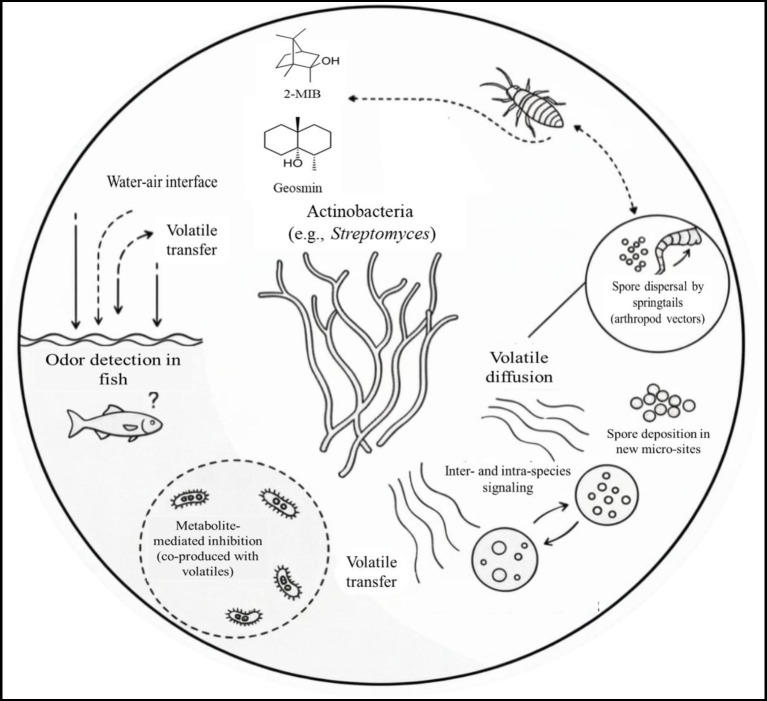
Ecological interaction network of geosmin and 2-MIB produced by Actinobacteria (Becher et al., 2020). Actinobacteria such as *Streptomyces* release geosmin and 2-MIB, which diffuse through soil and water and can cross the water–air interface to be detected by fish. These volatiles also attract springtails that transport spores to new microsites, enhancing dispersal. In parallel, geosmin and 2-MIB act as signaling molecules that mediate microbial communication and often accompany the production of inhibitory metabolites. Conceptual illustration generated with Google Gemini and edited by the authors.

The synthesis of geosmin and 2-methylisoborneol (2-MIB) by Actinobacteria such as *Streptomyces* and *Nocardia* illustrates the functional duality of these taxa rather than an inherently undesirable trait. In natural soils and aquatic sediments, these volatile terpenoids act as info chemicals that mediate interactions with invertebrates and other microorganisms. In ecological contexts where these volatiles are produced, geosmin and 2-MIB may attract arthropods that feed on microbial biomass and disperse spores, thereby promoting colonization of new microsites and supporting Actinobacterial persistence under strong competitive pressure (Becher et al., 2020; Garbeva et al., 2023). These volatiles form part of a broader signaling network acting in concert with non-volatile metabolites to influence community assembly, resource use, and nutrient cycling (Figure 10).

In intensive aquaculture systems, the same metabolites become problematic only when their concentrations exceed human sensory thresholds. Geosmin levels around or above 10 ng L^−1^, which are compatible with dispersal functions in natural environments, are sufficient to confer earthy or musty off-flavors in species such as *O. niloticus* and *O. mykiss*, thereby reducing market value without necessarily compromising fish health (Lindholm-Lehto et al., 2019; Lopes et al., 2022). Experimental studies show that geosmin and 2-MIB biosynthesis is tightly regulated by nutrient availability, oxygen conditions, and microbial interactions: nitrogen limitation in *S. luridiscabiei* increased geosmin and 2-MIB production by 300–500%, whereas reduced oxygen availability in *S. roseoflavus* decreased 2-MIB yields by 50–70% (Guttman and van Rijn, 2008; Schrader, 2020). Observed correlations between *Actinomycetales* abundance, *geoA* expression, and geosmin accumulation in recirculating aquaculture systems, together with potential modulation by myxobacterial signaling, further highlight the role of community structure and cross-kingdom communication in volatile output (Churro et al., 2020; Lukassen et al., 2019).

Crucially, the same microbial networks also harbour intrinsic remediation potential. Several non-Actinobacterial genera, such as *Pseudomonas*, *Flavobacterium,* and *Puccinia*, have been reported as effective degraders of 2-MIB and geosmin through oxidative and dehydrogenation pathways. Importantly, this degradative capacity is also present within the Actinobacterial lineage that produces these metabolites. Actinobacteria such as *Micrococcus*, *Rhodococcus erythropolis,* and *Rhodococcus xanthus* can efficiently degrade 2-MIB, indicating that Actinobacterial communities may contain both producers and degraders, enabling inherent attenuation of off-flavor compounds (Wang et al., 2025). Beyond microbial degradation, several system-level processes, such as biological activated carbon (BAC) filtration, granular activated carbon (GAC) adsorption, and controlled oxidative treatments (e.g., ozonation or UV-based processes), have been shown to reduce geosmin and 2-MIB without disrupting microbial community structure (Wang et al., 2023). This underscores that off-flavor accumulation is not an unavoidable outcome of Actinobacterial presence, but a manageable interaction between system design, environmental conditions, and the functional diversity of resident microbial communities.

Actinobacterial toxigenic potential must also be considered when selecting candidates for aquaculture applications. Reports of tetrodotoxin (TTX) associated with marine Actinobacteria in pufferfish tissues, including low levels in ovaries, indicate that microbial communities may contribute to toxin accumulation in some species (Wu et al., 2005). Although the biosynthetic origin of TTX and the role of horizontal gene transfer remain unresolved, these findings justify targeted monitoring in known high-risk taxa using sensitive analytical methods such as LC–MS/MS and immunoassays, rather than generalized concern across all Actinobacteria (Zhang et al., 2024).

Similarly, a few Actinobacterial lineages, such as *M. chesapeaki* and specific *Streptomyces griseus* isolates, have been confirmed as pathogenic in farmed fish (Heckert et al., 2001; Lewis et al., 2009). Their existence underscores the need for genome-resolved identification, virulence and toxin screening, and exclusion of such strains from probiotic or biocontrol pipelines (Stentiford et al., 2017). In this context, pathogenic and toxigenic cases do not discredit Actinobacteria but highlight the importance of rigorous, strain-level risk assessment when translating their metabolic diversity into aquaculture practice.

Taken together, these observations reinforce the notion of metabolic duality as an opportunity rather than a constraint. Actinobacteria provide a rich arsenal of bioactive molecules that can be harnessed for pathogen control and host support, while metabolites perceived as undesirable in certain production contexts can be mitigated through ecological engineering, strain selection, and informed management of microbial communities. The priority is not to exclude Actinobacteria from aquaculture systems but to characterize them with higher genomic and functional resolution, select strains aligned with specific health and quality objectives, and design conditions that favor metabolic benefits over detrimental effects.

## 5 Conclusion

Actinobacteria represent a microbial group of critical importance in contemporary aquaculture due to their dual functionality. They are distinguished by their capacity to synthesize antimicrobial metabolites with proven efficacy against relevant pathogens, alongside their potential to modulate immune responses in fish. On the other hand, these bacteria exhibit adverse characteristics, such as the biosynthesis of compounds responsible for organoleptic alterations, including geosmin and 2-methylisoborneol, as well as the production of toxins, such as tetrodotoxin, whose bioaccumulation dynamics remain insufficiently characterized. This duality reflects complex evolutionary adaptations within highly competitive microbial environments.

The scarcity of comprehensive genomic data for numerous strains impedes the precise characterization of their biosynthetic capabilities and regulatory mechanisms, posing a significant challenge to their controlled and safe application in aquaculture systems. Therefore, future studies should incorporate genome-resolved approaches and report genome accession numbers (e.g., NCBI/ENA/DDBJ) to ensure traceability, strain-level validation, and reproducibility in aquaculture probiotic research. Consequently, current understanding of the mechanisms governing metabolic production must rely on integrative approaches that incorporate environmental variables and microbial interaction dynamics.

The emerging frontier in aquaculture lies not in the indiscriminate exploitation of their bioactive potential, but in decoding the evolutionary principles governing their metabolic chemistry. This knowledge will enable the design of strategies that emulate these mechanisms under rigorous control, ensuring sustainable and safe applications in aquatic systems.

## Data Availability

The data supporting the conclusions of this study are included in the article and its Supplementary Material. The PRISMA checklist is provided as Supplementary Table S1. The extracted study dataset is provided as a Data Sheet, and the appraisal outputs are available as Supplementary Table S2 (in vivo Risk of Bias) and Supplementary Table S3 (in vitro Quality Checklist).
