# Supplementary material for: The dual role of Actinobacteria in aquaculture: a systematic review of metabolic benefits and detrimental effects
Source: Front Microbiol. 2026 Jun 10;17:1794932. doi: 10.3389/fmicb.2026.1794932 (PMC13293482; doi:10.3389/fmicb.2026.1794932)
Supplement: Supplementary file 3 [file Table_3.docx]

**Methodological Quality and Reproducibility (***In vitro* **studies)**

Instructions: Mark each item as Yes, No, Unclear, or NA. Suggested scoring: Yes=1; No/Unclear=0; NA does not contribute. Then report Score (0–10) and Overall quality: High (7–10), Moderate (4–6), Low (0–3).

| **Study ID** | **Title** | **Strain ID adequate (16S/genome + accession)** | **Culture conditions described** | **Controls (neg + pos)** | **Replication (bio/tech) reported** | **Inoculum / concentration standardized** | **Quantitative method validated (MIC/MBC/standard)** | **Dose/exposure characterized** | **Statistics + variability reported** | **Key parameters reproducible** | **Safety assessed if claiming beneficial (or NA)** | **Score (0-10)** | **Overall quality (High/Mod/Low)** |
| --- | --- | --- | --- | --- | --- | --- | --- | --- | --- | --- | --- | --- | --- |
| Abdelaziz_2024 | A novel metabolite Streptomyces coeruleorubidus exhibits antibacterial activity against Streptococcus agalactiae through modulation of physiological performance, inflammatory cytokines, apoptosis, and oxidative stress-correlated gene expressions in Nile tilapia | Yes | Yes | Unclear | Yes | Yes | Yes | Yes | Yes | Yes | Yes | 90% (9/10) | High |
| Klausen_2005 | Abundance of actinobacteria and production of geosmin and 2-methylisoborneol in Danish streams and fish ponds | Yes | Yes | Unclear | Yes | Yes | Yes | Yes | Yes | Yes | Yes | 90% (9/10) | High |
| Pan_2009 | Actinomycetes and earthy-musty odorous compounds in brackish fishponds in Tianjin, china | Unclear | Unclear | NA | Unclear | NA | Unclear | NA | Unclear | Unclear | NA | 0% (0/6) | Low |
| Zeng_2024 | Actinomycin D reduces virulence factors and biofilms against Aeromonas hydrophila | Yes | Yes | Unclear | Yes | Yes | Yes | Yes | Yes | Yes | Yes | 90% (9/10) | High |
| Selvan_2012 | Antagonistic activity of marine sponge associated Streptomyces sp. Against isolated fish pathogens | Yes | Yes | Unclear | Yes | Yes | Yes | Yes | Yes | Yes | Yes | 90% (9/10) | High |
| Kamarudheen_2015 | Antagonistic activity of marine Streptomyces sp. on fish pathogenic vibrio species isolated from aquatic environment | Yes | Yes | Unclear | Unclear | Yes | Yes | Yes | Yes | Yes | Unclear | 70% (7/10) | Moderate |
| Nabila_2018 | Antagonistic activity of terrestrial streptomyces sp. Vitnk9 against gram negative bacterial pathogens affecting the fish and shellfish in aquaculture | Yes | Yes | Yes | Yes | Yes | Yes | Yes | Yes | Yes | Unclear | 90% (9/10) | High |
| Boutin_2012 | Antagonistic effect of indigenous skin bacteria of brook charr (Salvelinus fontinalis) against Flavobacterium columnare and F. psychrophilum | Yes | Yes | Unclear | Yes | Yes | Yes | Yes | Yes | Yes | Unclear | 80% (8/10) | High |
| Dharmaraj_2011 | Antagonistic potential of marine Actinobacteria against fish and shellfish pathogens | Yes | Yes | Yes | Unclear | Yes | Yes | Yes | Yes | Yes | Yes | 90% (9/10) | High |
| Ravikumar_2012 | Antagonistic properties of seagrass associated Streptomyces sp. RAUACT-1: A source for anthraquinone rich compound | Yes | Yes | Unclear | Yes | Yes | Yes | Yes | Yes | Yes | NA | 89% (8/9) | High |
| Nabila_2018 | Antibacterial activity of soil actinomycetes against fish and shellfish pathogens | Yes | Yes | Unclear | Unclear | Yes | Yes | Yes | Yes | Yes | Yes | 80% (8/10) | Moderate |
| Cho_2012 | Antibacterial benzaldehydes produced by seaweed-derived Streptomyces atrovirens PK288-21 | Yes | Yes | NA | Yes | NA | Yes | NA | NA | Yes | NA | 100% (5/5) | High |
| Miller_2022 | Antibiofilm properties of bioactive compounds from Actinomycetes against foodborne and fish pathogens | Yes | Yes | Yes | Yes | Yes | Yes | Yes | Yes | Yes | NA | 100% (9/9) | High |
| Graa_2013 | Antimicrobial activity of heterotrophic bacterial communities from the marine sponge Erylus discophorus (Astrophorida, Geodiidae) | Yes | Yes | NA | Yes | NA | Yes | NA | NA | Yes | NA | 100% (5/5) | High |
| Elumalai_2024 | Bioactive compound from marine seagrass Streptomyces argenteolus TMA13: combatting fish pathogens with time-kill kinetics and live-dead cell imaging | Yes | Yes | Unclear | Yes | Yes | Yes | Yes | Yes | Yes | NA | 89% (8/9) | High |
| Kumaran_2020 | Bioactive metabolites produced from Streptomyces enissocaesilis SSASC10 against fish pathogens | Yes | Yes | Unclear | Yes | Yes | Yes | Yes | Yes | Yes | Yes | 90% (9/10) | High |
| Manisha_2019 | Bioprospecting of actinobacteria from the Andaman marine ecosystem: Isolation, antagonistic potential, and taxonomy of potential strain | Yes | Yes | Yes | Unclear | Yes | Yes | Yes | Yes | Yes | Yes | 90% (9/10) | High |
| Thirumurugan_2015 | Characterization and structure elucidation of antibacterial compound of Streptomyces sp. ECR77 isolated from east coast of India | Yes | Yes | Unclear | Yes | Yes | Yes | Yes | Yes | Yes | NA | 89% (8/9) | High |
| Zuo_2010 | Contribution of Streptomyces in sediment to earthy odor in the overlying water in Xionghe Reservoir, China | Yes | Yes | Unclear | Yes | Yes | Yes | Yes | Yes | Yes | Unclear | 80% (8/10) | High |
| Giro | Decylprodigiosin: a new member of the prodigiosin family isolated from a seaweed-associated Streptomyces | Yes | Yes | NA | Yes | NA | Yes | NA | NA | Yes | NA | 100% (5/5) | High |
| Schrader_2010 | Distribution of off-flavor compounds and isolation of geosmin-producing bacteria in a series of water recirculating systems for rainbow trout culture | Yes | Yes | NA | Yes | NA | Yes | NA | NA | Yes | NA | 100% (5/5) | High |
| Schrader_2001 | Effects of carbon source, phosphorus concentration, and several micronutrients on biomass and geosmin production by Streptomyces halstedii | Unclear | Unclear | Unclear | Unclear | Unclear | Yes | Unclear | Unclear | Unclear | NA | 11% (1/9) | Low |
| Schrader_2020 | Effects of relevant ammonium chloride concentrations on biomass and off-flavor compound production by Streptomyces luridiscabiei originating from a recirculating aquaculture system | Yes | Yes | Unclear | Yes | Unclear | Yes | Yes | Yes | Yes | NA | 78% (7/9) | High |
| Wu_2020 | Effects of woody forages on biodiversity and bioactivity of aerobic culturable gut bacteria of tilapia (Oreochromis niloticus) | Unclear | Unclear | Unclear | Yes | Unclear | Unclear | Unclear | Yes | Unclear | NA | 22% (2/9) | Low |
| Dharmaraj_2010 | Evaluation of Streptomyces as a probiotic feed for the growth of ornamental fish Xiphophorus helleri | Yes | Yes | Yes | Yes | Yes | Yes | Yes | Yes | Yes | Unclear | 90% (9/10) | High |
| Sanchez_2012 | Examining the fish microbiome: vertebrate-derived bacteria as an environmental niche for the discovery of unique marine natural products | Yes | Yes | NA | Yes | NA | Yes | NA | NA | Yes | NA | 100% (5/5) | High |
| Schrader_1993 | Geosmin-producing species of Streptomyces and Lyngbya from aquaculture ponds | Yes | Yes | Unclear | Yes | Yes | Yes | Yes | Yes | Yes | NA | 89% (8/9) | High |
| Guttman_2008 | Identification of conditions underlying production of geosmin and 2-methylisoborneol in a recirculating system | Yes | Yes | Unclear | Yes | Yes | Yes | Yes | Yes | Yes | NA | 89% (8/9) | High |
| Lukassen_2022 | Impact of water quality parameters on geosmin levels and geosmin producers in European recirculating aquaculture systems | Yes | Yes | Unclear | Yes | Unclear | Yes | Yes | Yes | Yes | NA | 78% (7/9) | High |
| Choudhury_2008 | Inhibition of actinomycetes to histamine producing bacteria associated with indian Mackerel fish (Rastrelliger kanagurta, Cuvier, 1816) | Unclear | Unclear | Unclear | Unclear | Unclear | Unclear | Unclear | Unclear | Unclear | NA | 0% (0/9) | Low |
| You_2007 | Inhibition of Vibrio biofilm formation by a marine actinomycete strain A66 | Yes | Yes | Yes | Unclear | Yes | Yes | Yes | Yes | Yes | NA | 89% (8/9) | High |
| Chakraborty_2015 | Isolation and characterization of antagonistic Streptomyces spp. From marine sediments along the southwest coast of India | Yes | Yes | Yes | Yes | Yes | Yes | Yes | Yes | Yes | NA | 100% (9/9) | High |
| Lauzon_2008 | Isolation of putative probionts from cod rearing environment | Yes | Yes | Yes | Yes | Yes | Yes | Yes | Yes | Yes | Unclear | 90% (9/10) | High |
| Thirumurugan_2018 | Isolation, structure elucidation and antibacterial activity of methyl-4,8-dimethylundecanate from the marine actinobacterium Streptomyces albogriseolus ECR64 | Yes | Yes | Unclear | Yes | Yes | Yes | Yes | Yes | Yes | Yes | 90% (9/10) | High |
| Jami_2015 | Phylogenetic diversity and biological activity of culturable Actinobacteria isolated from freshwater fish gut microbiota | Yes | Unclear | Unclear | Yes | Yes | Yes | Yes | Yes | Unclear | Unclear | 60% (6/10) | Moderate |
| Long_2024 | Streptomyces enissocaesilis L-82 has broad-spectrum antibacterial activity and promotes growth for Carassius auratus | Yes | Yes | Yes | Yes | Yes | Yes | Yes | Yes | Yes | Yes | 100% (10/10) | High |
| Ali_2013 | Studies on actinomycetes collected from pond sediment inhibiting fish pathogenic and human clinical bacterial isolates | Unclear | Unclear | Unclear | Unclear | Unclear | Unclear | Unclear | Unclear | Unclear | NA | 0% (0/9) | Low |
| Essawy_2021 | Synergistic Effect of Biosynthesized Silver Nanoparticles and Natural Phenolic Compounds against Drug-Resistant Fish Pathogens and Their Cytotoxicity: An In Vitro Study | Yes | Yes | Yes | Yes | Unclear | Yes | Yes | Yes | Yes | Yes | 90% (9/10) | High |
| Do_1991 | Tetrodotoxin production of actinomycetes isolated from marine sediment | Unclear | Unclear | Unclear | Unclear | Unclear | Unclear | Unclear | Unclear | Unclear | NA | 0% (0/9) | Low |
| Wu_2005 | Toxicity and distribution of tetrodotoxin-producing bacteria in puffer fish Fugu rubripes collected from the Bohai Sea of China | Yes | Yes | Unclear | Yes | Yes | Yes | Yes | Yes | Yes | Yes | 90% (9/10) | High |
| Neu_2014 | Toxicity of bioactive and probiotic marine bacteria and their secondary metabolites in Artemia sp. and Caenorhabditis elegans as eukaryotic model organisms | Yes | Yes | Yes | Yes | Yes | Yes | Yes | Yes | Yes | Yes | 100% (10/10) | High |
| Sharma_2020 | Two antibacterial and PPARÎ±/Î³-agonistic unsaturated keto fatty acids from a coral-associated actinomycete of the genus <i>Micrococcus</i> | Yes | Yes | Yes | Yes | Yes | Yes | Yes | Yes | Yes | Yes | 100% (10/10) | High |

**Supplementary Table S2. In vitro quality appraisal and reproducibility checklist for included studies.**Quality assessment of included in vitro studies using a prespecified reproducibility-focused checklist. Items capture strain/compound identification, culture conditions, controls, replication, inoculum/exposure standardization, quantitative endpoints, and reporting of variability/statistics. Items were rated as Yes/No/Unclear/Not applicable (NA), and an overall quality score was calculated as the percentage of “Yes” across evaluable items (excluding NA). Studies were categorized as High/Moderate/Low quality using prespecified thresholds. Assessments were performed independently by two reviewers, with discrepancies resolved by consensus.
