# Supplementary material for: The dual role of Actinobacteria in aquaculture: a systematic review of metabolic benefits and detrimental effects
Source: Front Microbiol. 2026 Jun 10;17:1794932. doi: 10.3389/fmicb.2026.1794932 (PMC13293482; doi:10.3389/fmicb.2026.1794932)
Supplement: Supplementary file 4 [file Table_4.docx]

**Risk of Bias Assessment (***In vivo* **studies)**

Instructions: For each domain, mark Low, High, or Unclear risk of bias (use NA when not applicable). Then assign Overall RoB (Low / Some concerns / High).

| **Study ID** | **Title** | **Design** | **Randomization** | **Allocation concealment** | **Blinding (outcome)** | **Unit of analysis (tank vs fish)** | **Baseline/conditions comparable** | **Incomplete outcome data** | **Selective reporting** | **Co-interventions controlled** | **Other bias (e.g., challenge standardization)** | **Overall RoB** |
| --- | --- | --- | --- | --- | --- | --- | --- | --- | --- | --- | --- | --- |
| Abdelaziz_2024 | A novel metabolite Streptomyces coeruleorubidus exhibits antibacterial activity against Streptococcus agalactiae through modulation of physiological performance, inflammatory cytokines, apoptosis, and oxidative stress-correlated gene expressions in Nile tilapia | Interventional/Challenge | Low | Unclear | Unclear | Low | Low | Low | Unclear | Low | Unclear | Low |
| Zeng_2024 | Actinomycin D reduces virulence factors and biofilms against Aeromonas hydrophila | Interventional (insect survival assay) | Unclear | NA | Unclear | Low | Unclear | Low | Unclear | Low | Unclear | Some concerns |
| Boutin_2012 | Antagonistic effect of indigenous skin bacteria of brook charr (Salvelinus fontinalis) against Flavobacterium columnare and F. psychrophilum | Interventional/Natural outbreak | Unclear | NA | Unclear | High | Unclear | Low | Unclear | Unclear | High | High |
| Heckert_2001 | Detection of a new Mycobacterium species in wild striped bass in the Chesapeake bay | Observational (case series/field sampling) | NA | NA | NA | NA | High | Unclear | Unclear | NA | High | High |
| Dharmaraj_2010 | Evaluation of Streptomyces as a probiotic feed for the growth of ornamental fish Xiphophorus helleri | Feeding trial | Unclear | NA | Unclear | High | Unclear | Unclear | Unclear | Low | High | High |
| Long_2024 | Streptomyces enissocaesilis L-82 has broad-spectrum antibacterial activity and promotes growth for Carassius auratus | Feeding trial | Low | Unclear | Unclear | Low | Low | Low | Unclear | Low | Unclear | Low |
| Neu_2014 | Toxicity of bioactive and probiotic marine bacteria and their secondary metabolites in Artemia sp. and Caenorhabditis elegans as eukaryotic model organisms | Interventional toxicity assay (invertebrate models) | Unclear | NA | Unclear | Unclear | Low | Low | Unclear | Low | Low | Some concerns |
| Lewis_2008 | Toxic effects of Streptomyces griseus spores and exudate on gill pathology of freshwater fish | Interventional exposure trial (lab, multi-species) | Unclear | NA | Unclear | Unclear | Low | Low | Unclear | Low | Low | Some concerns |

**Supplementary Table S2. *In vivo* risk of bias assessment of included studies.** Domain-based risk-of-bias ratings for each included in vivo study. Domains include randomization/allocation concealment, blinding, unit-of-analysis (fish vs tank/experimental unit), incomplete outcome data, selective reporting, and other potential sources of bias. Each domain was rated as Low/Unclear/High risk, and an overall judgment (Low/Some concerns/High) was assigned using prespecified decision rules. Assessments were performed independently by two reviewers, with discrepancies resolved by consensus
